# In Situ Microscopy Analysis Reveals Local Innate Immune Response Developed around *Brucella* Infected Cells in Resistant and Susceptible Mice

**DOI:** 10.1371/journal.ppat.1002575

**Published:** 2012-03-29

**Authors:** Richard Copin, Marie-Alice Vitry, Delphine Hanot Mambres, Arnaud Machelart, Carl De Trez, Jean-Marie Vanderwinden, Stefan Magez, Shizuo Akira, Bernhard Ryffel, Yves Carlier, Jean-Jacques Letesson, Eric Muraille

**Affiliations:** 1 Unité de Recherche en Biologie Moléculaire, Laboratoire d'Immunologie et de Microbiologie, Faculté Universitaire Notre Dame de la Paix, Namur, Belgium; 2 Department of Molecular and Cellular Interactions, Vlaams Interuniversitair Instituut voor Biotechnologie, Vrije Universiteit Brussel, Brussels, Belgium; 3 Laboratoire de Neurophysiologie, Faculté de Médecine, Université Libre de Bruxelles, Bruxelles, Belgium; 4 Department of Host Defense, Research Institute for Microbial Diseases, Osaka University Yamadaoka, Suita City, Osaka, Japan; 5 University of Orleans, Transgenose Institute, Laboratoire d'Immunologie et d'Embryologie Moléculaires, CNRS, UMR 6218, Orleans, France; 6 Laboratoire de Parasitologie, Faculté de Médecine, Université Libre de Bruxelles, Bruxelles, Belgium; Yale University School of Medicine, United States of America

## Abstract

*Brucella* are facultative intracellular bacteria that chronically infect humans and animals causing brucellosis. *Brucella* are able to invade and replicate in a broad range of cell lines *in vitro*, however the cells supporting bacterial growth *in vivo* are largely unknown. In order to identify these, we used a *Brucella melitensis* strain stably expressing mCherry fluorescent protein to determine the phenotype of infected cells in spleen and liver, two major sites of *B. melitensis* growth in mice. In both tissues, the majority of primary infected cells expressed the F4/80 myeloid marker. The peak of infection correlated with granuloma development. These structures were mainly composed of CD11b^+^ F4/80^+^ MHC-II^+^ cells expressing iNOS/NOS2 enzyme. A fraction of these cells also expressed CD11c marker and appeared similar to inflammatory dendritic cells (DCs). Analysis of genetically deficient mice revealed that differentiation of iNOS^+^ inflammatory DC, granuloma formation and control of bacterial growth were deeply affected by the absence of MyD88, IL-12p35 and IFN-γ molecules. During chronic phase of infection in susceptible mice, we identified a particular subset of DC expressing both CD11c and CD205, serving as a reservoir for the bacteria. Taken together, our results describe the cellular nature of immune effectors involved during *Brucella* infection and reveal a previously unappreciated role for DC subsets, both as effectors and reservoir cells, in the pathogenesis of brucellosis.

## Author Summary

*Brucella* are facultative intracellular bacteria chronically infecting humans and animals causing brucellosis, one of the most common zoonotic disease worldwide which can result in infertility and chronic debilitating disease. The cells supporting *Brucella* growth *in vivo* remain largely unknown. In order to identify these, we constructed a *Brucella melitensis* strain expressing a fluorescent protein that allowed us to characterize infected cells by microscopy of the spleen and liver from infected mice. In both tissues, the majority of primary infected cells were cells from the macrophage lineage. The peak of infection correlated with granuloma development. These structures contained the majority of bacteria and were mainly composed of cells expressing CD11b, F4/80, MHC-II, which are specific of activated monocytes/macrophages. A fraction of granuloma cells also expressed CD11c and were similar to inflammatory dendritic cells (DCs). During the chronic phase of infection in susceptible mice, we identified a particular subset of DC expressing CD205 and serving as a reservoir for the bacteria. Overall, our results describe the nature of immune cells infected by *Brucella in vivo* and reveal an unappreciated role for DC subsets, both as effectors and reservoir cells. These results could help develop new therapeutic strategies to control *Brucella* infection.

## Introduction

*Brucella* (α-proteobacteria) are facultative intracellular Gram-negative coccobacilli that infect humans as well as domestic (goat, sheep, swine, etc.) and wild type mammals. Animal infection leads to abortion and infertility with dramatic economic costs. Brucellosis is mainly transmitted to humans through the ingestion of raw milk or non-pasteurized cheese contaminated with *Brucella*. It is characterized by undulant fever which, if left untreated, can result in chronic disease with serious clinical manifestations, such as orchitis, osteoarthritis, spondylitis, endocarditis, and several neurological disorders [1]–[5]. Human brucellosis remains a significant public health concern in areas of the world where *Brucella* infections are endemic in food animals. Indeed, brucellosis has been described as being the most common zoonotic disease worldwide with more than 500,000 new human cases annually [4].

*Brucella* are highly infectious via oral and aerosol routes and difficult to treat with antibiotics. No safe or effective vaccine is available to prevent human infection. These characteristics justify the classification of *Brucella* strains as a category B pathogens, which represent a risk for use as bioweapons [6],[7].

*B. melitensis* is the most frequent cause of human brucellosis [1], [8]. Recently, bioluminescent *B. melitensis* has been used to visualize the dynamic of bacterial dissemination following intraperitoneal (i.p.) inoculation in mice [9]. Results confirmed that this model parallels human infection and identified major sites of bacterial growth, such as the spleen and the liver, during the early chronic phase of infection. However, cells supporting bacterial growth *in vivo* in these organs and serving as reservoir are unknown.

Despite recent progress in mouse models of brucellosis, much remains unknown regarding cellular components of the innate and adaptive immune responses induced by *B. melitensis* infection. We and others have shown that IFN-γ-producing CD4^+^ T cells [10]–[14] and inducible Nitric Oxide Synthase (iNOS/NOS2)-producing inflammatory dendritic cells (iNOS-DC) [10] are major components of protective immune response against *B. melitensis*. Activation of these cells involves Toll Like Receptor (TLR) 4 and TLR9 coupled to MyD88 adaptor protein [10]. However, there is little understanding on where these cells are localized *in situ* so they can initiate cellular interactions to control infection. Several experimental models of infection by intracellular bacteria such as *Mycobacteria tuberculosis* [15] and *Listeria monocytogenes* [9], [16] have illustrated the importance of the granulomatous lesion in limiting both tissue damage and bacterial dissemination. The granulomatous lesion is an organized and dynamic structure implicating activated monocytes surrounded by T cells and granulocytes. Its formation involves an orchestrated production of chemokines and cytokines and the upregulation of their cognate receptors along with the expression of addressins, selectins and integrins. Altogether, these elements coordinate the recruitment, migration and retention of cells to and within the granuloma. Chronic granulomatous inflammation has been reported in spleen and liver from natural hosts, human and mice infected by *Brucella* bacteria [17]. However, the importance of granulomas in the control of *Brucella* growth, their cellular composition and the signalling pathways implicated in their formation are largely unknown.

To address these issues, we developed a *B. melitensis* strain stably expressing mCherry fluorescent protein (mCherry-Br). This novel tool has allowed us to determine the phenotype of *Brucella*-targeted cells and characterize the composition and the dynamic of granuloma in the spleens and livers of infected mice. We observed the formation of iNOS^+^ granuloma structures surrounding infected cells and identified iNOS-DC (CD11b^+^ CD11c^+^ Ly-6G^−^ MHC-II^+^ iNOS^+^) and activated monocytes (CD11b^+^ CD11c^−^ Ly-6G^−^ MHC-II^+^ iNOS^+^) as the major cell types constituting granulomatous lesions in both tissues. In addition, we observed that alteration of the MyD88/IL-12/IFN-γ axis deeply affects the cellular composition of granulomas, reducing their ability to control bacterial dissemination and leading to replication and persistence of the pathogen in distinct cellular niches.

## Results

### Construction of mCherry stably expressing *Brucella melitensis* 16M

In order to detect infected cells on tissue sections by fluorescent microscopy, we constructed a constitutively fluorescent strain of *B. melitensis*. The mCherry protein, a previously described rapidly maturing variant of the red fluorescent protein DsRed [18], was cloned into the suicide vector *pKSoriT-bla-kan* downstream of a strong *Brucella* spp. promoter P*sojA* [19], [20]. The final construct was transformed into *Escherichia coli* strain S17-1, and introduced into *B. melitensis* strain by conjugation (see Material and Method for details).

Flow cytometry (Figure S1.A) and fluorescent microscopy (Figure S1.B) were used to validate mCherry-expressing *Brucella* (mCherry-Br) strain *in vitro*. The comparison of wild type and mCherry-Br strains growth curve revealed that mCherry gene expression does not affect ability of *B. melitensis* to replicate *in vitro* (data not shown). In addition, following i.p. inoculation of 4×10^4^ CFU, we showed that mCherry-Br was able to infect chronically resistant C57BL/6 mice (Figure S1.C). At 5 days post infection (p.i.), bacterial burden in spleen from mCherry-Br infected mice was reduced by approximately half-a-log when compared to wild type bacteria-infected mice. However, at 25 and 42 days p.i., bacterial counts from both groups were similar, suggesting that bacterial virulence is moderately and transiently affected by the insertion of mCherry tracer.

### Detection of mCherry-expressing *Brucella* in tissues from infected mice

In order to characterize the spatio-temporal behavior of *Brucella* in spleen and liver of infected animals, we used immuno-fluorescence microscopy techniques. At low dose of infection (10^4^ to 10^6^ CFU) in C57BL/6 and BALB/c wild type mice, growth of *B. melitensis* peaked at 5 days p.i. with modest CFU counts (between 10^5^–10^6^ CFU/g of spleen [10]) rendering its visualization on tissue sections very difficult. Our preliminary studies demonstrated that 10^6^ CFU/g of tissue was the limit of sensitivity of our technique, with less than 0.1 infected cells by observation surface (200×230 µm) in tissue sections of 5 µm in thickness and using a 63× objective (data not shown). Therefore, in order to maximize our chances of visualizing the dynamic behavior of mCherry-Br *in vivo* several days after inoculation, we injected 10^8^ CFU of bacteria in wild type mice. With this dose, no increase in mortality of mCherry-Br infected wild type C57BL/6 and BALB/c mice was observed (data not shown). Importantly, when compared to the routinely used low bacterial inoculum, a high infectious dose (10^8^ CFU) showed similar impact on the composition of recruited spleen cell populations involved in innate inflammatory responses to *Brucella*. Overall, a high bacterial inoculum only shorten the kinetic of cell recruitment (Figure S2).

First analyses revealed that ten minutes after i.p. inoculation, bacteria were massively present in the spleen and the liver, with an average of 10^6^ CFU/g (Figure S3.A). Both organs maintained a level of 10^6^–10^8^ CFU/g during the 5 first days p.i. (Figure S3.A), allowing the analysis of mCherry-Br infected cells *in situ* during this time (Figure S3.B). Infected susceptible BALB/c mice displayed significantly higher count of bacteria in the spleen at 3 and 5 days p.i. when compared to C57BL/6 mice. However, at day 12, spleens and livers from wild type mice had less than 10^5^–10^6^ CFU/g and bacteria became undetectable by microscopy analysis (data not shown). Consequently, we limited our analysis to 120 h p.i. in wild type mice.

### First *Brucella*-infected splenocytes are mainly F4/80^+^ red pulp macrophages and MOMA-1^+^ metallophilic marginal zone macrophages

The functions of the spleen are centered on systemic blood circulation. As such, it lacks afferent lymphatic vessels. It is comprised of two functionally and morphologically distinct compartments, namely the red pulp (r.p.) and the white pulp (w.p.). The r.p. is a blood filter rich in F4/80^+^ macrophages that removes foreign material as well as damaged and effete erythrocytes. The spleen is also the largest secondary lymphoid organ of the body that initiates immune responses to blood-borne antigens. This function is usually associated to the w.p. compartment. This latter is centered on the central arteriole within the T cell rich area surrounded by B lymphocyte-associated follicles. At the interface between the r.p. and w.p., the marginal zone (m.z.) is a unique region of the spleen. Considered to be a separate compartment rather than part of the w.p., it is designed to screen systemic blood circulation for antigens and pathogens and plays an important role in antigen processing. It also contains several populations of specialized macrophages, such as marginal zone-associated metallophilic macrophages that can be identified with anti-MOMA-1 antibody (for a review see [21]).

**Figure 1:**
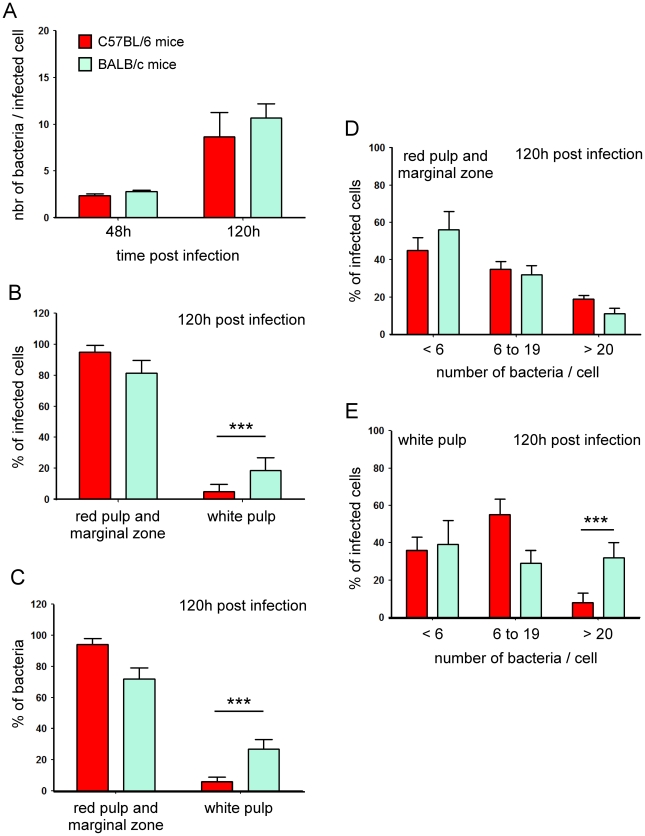
Spatial distribution of infected cells in spleen of C57BL/6 and BALB/c mice. Wild-type C57BL/6 and BALB/c mice were injected i.p. with PBS or 10^8^ CFU of mCherry-Br. Mice were sacrificed at selected times and spleens were collected and examined by immunohistofluorescence. **A**, The average number of bacteria per infected spleen cells at 48 h and 120 h p.i.. **B**, **C**, The percentage of infected cells and bacteria localized in the r.p. and m.z., or w.p. at 120 h p.i.. **D**–**E**, The comparative analysis of the bacterial load per cell present in the r.p. and m.z., or w.p.. For each conditions, a minimum of 200 cells has been examined. The bars are the mean±SD from at least 3 spleen sections per spleen from 6 mice.

**Figure 2:**
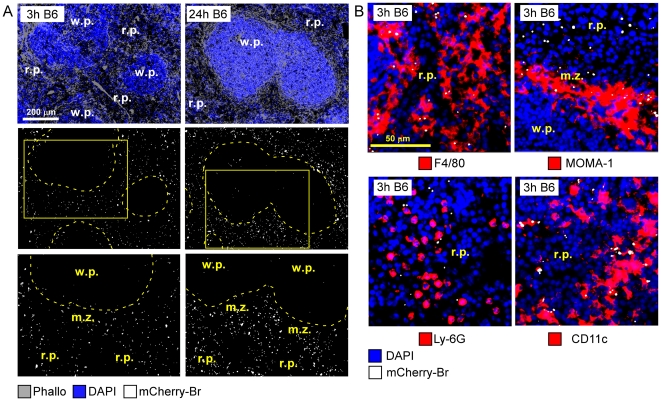
Red pulp localization of *B. melitensis* at the onset of infection. Wild-type C57BL/6 mice were injected i.p. with PBS or 10^8^ CFU of mCherry-Br. Mice were sacrificed at selected times and spleens were collected and examined by immunohistofluorescence. **A**, Positioning of mCherry-Br in the spleen of mice at 3 and 24 h p.i. The inset areas in the middle panels are shown in the bottom panels. **B**, Immunoflurescence analysis of F4/80, MOMA-1, Ly-6G, and CD11c expressing cells and mCherry-Br in the spleen of mice at 3 h p.i. Panels are color-coded with the text for the antigen or mCherry-Br examined. Scale bar = 200 and 50 µm, as indicated. r.p.: red pulp; w.p.: white pulp; m.z.: marginal zone. Data are representative of at least 3 independent experiments.

Following i.p. inoculation of 10^8^ CFU in resistant wild type C57BL/6 mice, mCherry-Br was detectable in the spleen by 10 minutes p.i. (Figure S3.A). Importantly, at this time, our observation showed that mCherry-Br already localized in the r.p. and the m.z. of infected spleens (data not shown). Then, bacteria increased exponentially during the first 24 h (Figure S3.A) and the number of intracellular bacteria peaked at 120 h p.i. with an average of 8–10 bacteria per cell (Figure 1.A). Importantly, infected cells were mainly located in m.z. and r.p. during the whole kinetic of infection (Figure 1.B–C and Figure 2.A) while infected cells were rarely observed in splenic w.p. area (w.p.).

**Figure 3:**
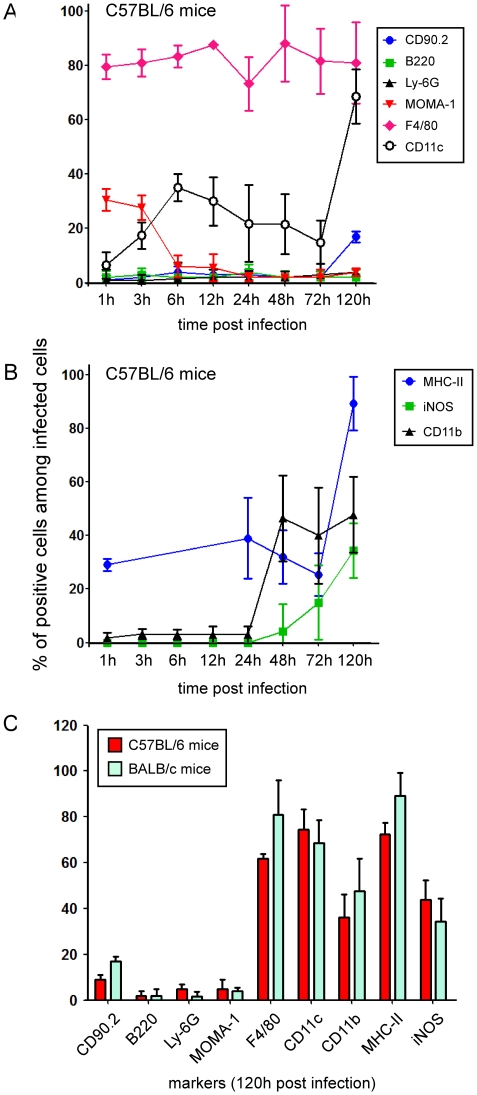
Phenotypical characterization of *B. melitensis* infected cells in the spleen. Wild-type C57BL/6 and BALB/c mice were injected i.p. with PBS or 10^8^ CFU of mCherry-Br. Mice were sacrificed at selected times and spleens were collected and examined by immunohistofluorescence. Data indicate the percentage of mCherry-Br that colocalizes with (**A**) CD90.2-, B220-, Ly6G-, MOMA-1-, F4/80-, CD11c-, (**B**) MHCII-, iNOS-, CD11b-expressing cells at the selected time in C57BL/7 mice. (**C**) Comparison at 120 h p.i. between C57BL/6 and BALB/c mice. The bars are the mean±SD from at least 3 spleen sections per spleen from 5 mice.

Further semi-quantitative analyses of infected cell phenotypes in C57BL/6 mice (Figure 2.B and Figure 3.A–B) showed that first infected cells in spleen were mainly r.p. macrophages with a F4/80^+^ CD11b^−^ phenotype (∼80%) and MOMA-1^+^ m.z. metallophilic macrophages (∼20%). Between 3 and 6 h after inoculation, the frequency of infected MOMA-1^+^ cells decreased strongly and CD11c^+^ dendritic cells progressively became infected (∼30% of total infected cells at 6 h) (Figure 3.A).

Using a home-made rabbit polyclonal antibody specific of *B. melitensis* (*Brucella*-Ag), we showed that mCherry-Br fluorescence and *Brucella*-Ag staining were generally overlapping (Figure S4), although there were some instances where *Brucella*-Ag staining could be detected in the absence of appreciable mCherry-Br fluorescence, possibly as a consequence of bacterial death or trafficking of antigens away from the bacteria. These results confirm that the majority of *Brucella*-infected cells can be detected *in situ* using mCherry-Br.

Confocal analysis in Z-stack demonstrated that bacteria localized inside F4/80^+^ r.p. macrophages (Figure S5.A–B). Importantly, this was also true for mCherry-expressing VirB-defective *Brucella* mutant (Figure S5.C). The VirB mutant displays a strongly reduced ability to infect cells *in vitro* [22] though it is able to persist several days *in vivo* and colonize mice similarly to wild type bacteria [23]. Therefore, it has been hypothesized that *in vivo* persistence could be due to extracellular replication [24]. However, our observations are not in favor of this hypothesis since VirB mutant and wild type bacteria both localized within cells of the same phenotype in infected spleen at least in the first 72 h p.i. (data not shown). As expected, VirB mutant is no longer detectable *in situ* at 120 h p.i.

### Control of *Brucella* infection in the spleen is correlated to the formation of F4/80^+^ MHC-II^+^ iNOS^+^ granulomas

**Figure 4:**
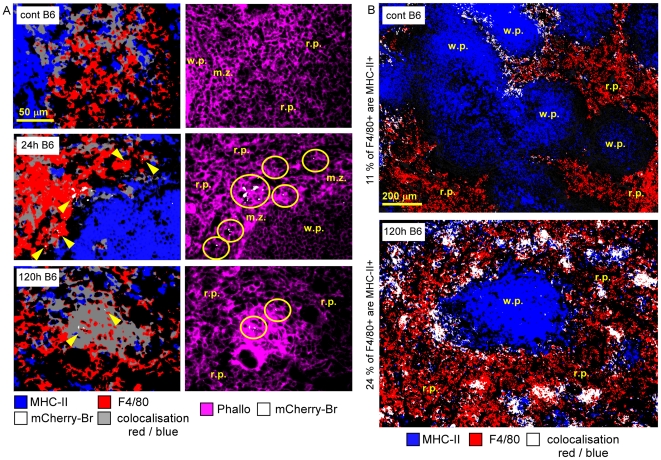
Characterization of F4/80^+^ infected cells in the spleen. Wild-type C57BL/6 mice were injected i.p. with PBS or 10^8^ CFU of mCherry-Br. Mice were sacrificed at selected times and spleens were collected and examined by immunohistofluorescence. **A**, Immunoflurescence analyses of F4/80, MHC-II, and actin expression in spleen of mice at 24 h and 120 h after mCherry-Br infection. Yellow circles and arrowheads indicate the presence of the bacterium. Panels are color-coded with the text for the antigen or mCherry-Br examined as well as the colocalization. **B**, Colocalization analysis of F4/80− and MHCII-expressing cells in spleen of mice before and 120 h after mCherry-Br infection. Scale bar = 50 and 200 µm, as indicated. r.p.: red pulp; w.p.: white pulp, m.z.: marginal zone. Data are representative of at least 3 independent experiments.

Our analysis revealed that the phenotype of infected spleen cells presented a drastic evolution between 24 h and 120 h p.i. Indeed, at 24 h p.i., about 80% of infected cells were CD11b^−^ F4/80^+^ r.p. macrophages (Figure 3.A–B), a fraction of which also expressed MHC-II^+^ molecules (Figure 3.B and Figure 4.A). Between 24 h and 72 h, the percentage of CD11b^+^ Ly-6G^−^-infected monocytes increased progressively (Figure 3.B and Figure S6). At 120 h, these monocytes constituted ∼30–40% of the infected cell population (Figure 3.B) and were mainly found clustered in granulomas (data not Shown).

**Figure 5:**
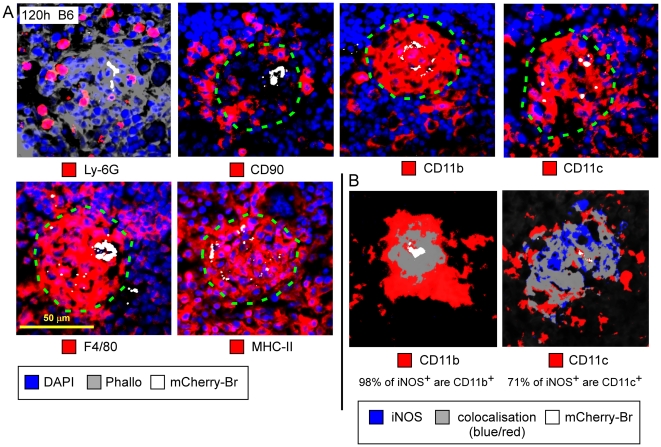
Phenotypical description of *B. melitensis* infected granulomas in the spleen. Wild-type C57BL/6 mice were injected i.p. with PBS or 10^8^ CFU of mCherry-Br. Mice were sacrificed at 120 h p.i. and spleens were collected and examined by immunohistofluorescence. **A**, Immunofluorescence analysis of Ly-6G, CD90.2, CD11b, CD11c, F4/80, and MHC-II expressing cells and mCherry-Br. **B**, Percentage of iNOS^+^ cells that colocalize with CD11b-, and CD11c-expressing cells. Numbers in B indicate the percentage of colocalizing cells in the upper panel. Images represent a single granuloma. Panels are color-coded with the text for the antigen or mCherry-Br examined as well as the colocalization. Scale bar = 50 µm, as indicated. Data are representative of at least 3 independent experiments.

Granulomas were constituted of CD11b^+^ F4/80^+^ Ly-6G^−^ MHC-II^+^ cells surrounded by CD90^+^ cells (T cells) and Ly-6G^+^ cells (granulocytes) (Figure 3.A–B, Figure 4.B, Figure 5.A). A fraction of CD11b^+^ F4/80^+^ MHC-II^+^ cells was also CD11c^+^ (Figure 5.A), suggesting that these cells were inflammatory DC. In previous reports, these inflammatory DC were detected by flow cytometry and identified as the main iNOS-producing cells during *Brucella* infection [10]. In agreement with this finding, we observed *in situ* that CD11b^+^ and CD11c^+^ cells located in granulomas were iNOS^+^ (Figure 5.B) and co-localized with mCherry-Br signal. Importantly, B cells (B220^+^ cells), T cells (CD90.2^+^ cells) or granulocytes (Ly-6G^+^ cells) seemed very rarely infected during the course of infection in the spleen (Figure 3.A–B and data not shown). Of note, a weak expression of CD90.2 was detected on a fraction of highly infected cells within granulomas (Figure 3.A). These cells displayed a cellular morphology (large cells with uncondensed nuclei) distinct from T lymphocytes and co-localized with CD11b and F4/80 (data not shown), suggesting that they were not lymphocytes but rather myeloid cells.

In summary, we concluded that infection seems largely limited to a fraction of the myeloid cell compartment in the spleen. Since the establishment of *Brucella* infection at day 5 in the spleen correlated with monocytes recruitment and formation of granulomas, we hypothesize that these latter are involved in bacterial containment and/or elimination as these structures contain most of the iNOS-producing cells. Granulomas were also detected in r.p. area of spleen from C57BL/6 mice infected for 5 days with 4×10^4^ CFU (Figure S7.A). As expected, their numbers were reduced when compared to mice infected with 10^8^ CFU. Surprisingly, at 12 days p.i. with both doses, granulomas were mostly located in w.p. area, specifically in T cells zones (Figure S7.A–B). Unfortunately, at this time point, we were not able to determine the presence of *Brucella* since the bacterial load was under our limit of detection. However, the similarity of the phenotype of these structures (CD11b^+^ and F4/80^+^, Figure S7.A and S7.B) with r.p. granulomas suggests that they may surround rare infected cells.

### *Brucella* infects similar cell phenotypes in resistant C57BL/6 and susceptible BALB/c mice

It has been frequently reported that BALB/c mice display higher bacterial loads per organs when compared to C57BL/6 mice during *Brucella* infection [10], [25]. The reasons for this susceptibility are unclear but it has been associated with lower frequency of IFN-γ-secreting CD4^+^ cells and iNOS-producing cells in these mice [10]. In accordance with CFU counts measured in spleens (Figure S3.A), the average number of bacteria per cell observed by microscopy analysis in BALB/c and C57BL/6 mice was similar at 48 h, though this number was slightly higher at 120 h in BALB/c mice (Figure 1.A). To determine whether enhanced susceptibility of BALB/c mice could be associated with the persistence of bacteria in distinct cell reservoirs, we further examined the phenotype of infected cells in both mouse strains at that time point. As observed for C57BL/6 mice, the large majority of infected cells were mainly located in splenic r.p. and m.z. in BALB/c mice (Figure 1.B–C) and semi-quantitative analyses of infected cell phenotypes (Figure 3.C) showed a global similarity between C57BL/6 and BALB/c mice. However, the frequency of infected cells located in w.p. was higher in BALB/c mice when compared to C57BL/6 mice (Figure 1.B–C). Further comparative analysis (Figure 1.D–E) of the spatial distribution of infected spleen cells showed that highly infected cells (>20 bacteria/cell) were more frequent in BALB/c infected mice and located mainly in w.p. areas.

In conclusion, infected cell phenotypes are relatively similar in both mouse strains but higher colonization of w.p. cells in infected BALB/c mice seems to explain for their higher CFU counts. These cells frequently expressed MOMA-1 or CD11c markers but were negative for CD11b, F4/80, Ly-6G and CD90.2 (data not shown).

### *Brucella* infection induces splenic dendritic cell migration and maturation

*Brucella* have frequently been described as silent or stealthy pathogens, able to actively escape the immune response through their ability to grow furtively within cells [26]. This hypothesis is mainly based on the fact that *Brucella*'s lipopolysaccharide (LPS) is a weak activator of macrophages and DC by comparison to conventional LPS from *Escherichia coli* [27]. In our study, we observed that CD11c^+^ DC represent 10–30% of spleen cells infected by mCherry-Br during the first 12 h of infection (Figure 2.B and Figure 3.A). Therefore, in our experimental conditions (10^8^ CFU i.p.), we investigated the impact of *Brucella* infection on DC activation *in vivo* in C57BL/6 mice. Imaging analysis (Figure S8) of spleen sections revealed that 24 h post inoculation, CD11c^high^ DCs relocated massively to w.p. T cell area.

Flow cytometry analyses demonstrated that DC migration observed *in situ* at 24 h p.i. was associated with their maturation as shown by cell surface up-regulation of MHC-II and CD86 co-stimulatory molecule expression on CD11c^high^ spleen cells (Figure S9.A). Although we observed maturation of all splenic DC subsets (CD8^−^ and CD8^+^) (data not shown), we also found that direct infection of DC was not required to trigger the phenomenon since DC observed by microscopy in w.p. T cell area at 24 h p.i. were not associated with mCherry-Br signal (data not shown). A lower dose of bacteria (4×10^4^ CFU) was able to induce DC maturation but the peak of maturation was reduced and displaced to 48 h p.i. (data not shown). Importantly, administration of heat-killed (HK) *Brucella* was also able to induce a dose-dependent DC maturation (Figure S9.B), suggesting that *Brucella*'s pathogen-associated molecular patterns (PAMPs) were sufficient to trigger this process. By comparison, HK *Escherichia coli* induced DC maturation at a ten-fold lower dose (Figure S9.B). Finally, using genetically deficient C57BL/6 mice, we investigated the role of MyD88 and TRIF adaptor molecules in DC maturation process *in vivo* and found that MyD88, but not TRIF, was important to *Brucella*-induced DC maturation (Figure S9.C).

### Granuloma formation is observed in *Brucella*-infected livers

Since the liver constitutes another important site of bacterial growth, we investigated the phenotype of infected cells in this organ in order to identify the common characteristics of infected cells in immunological and non-immunological sites. At 10 min p.i., mCherry-Br signal was already detectable in liver sections from infected C57BL/6 and BALB/c wild type mice and co-localized strictly with F4/80^+^ MHC-II^+^ Kupffer cells (data not shown). At 120 h post-inoculation, microscopy analyses revealed that, as observed in the spleen, the liver also displayed numerous granulomas composed of F4/80^+^ MHC-II^+^ CD11b^+^ cells (Figure S10). Granulomas frequently surrounded portal space and harbored the majority of infected cells (data not shown). As it was the case in the spleen, iNOS expression was mainly associated with a fraction of granuloma cells expressing CD11c, suggesting that activated monocytes and inflammatory DC were also major populations of these structures. Importantly, we never observed bacteria inside hepatocytes or Ly-6G^+^ granulocytes suggesting that, like in the spleen, infection is limited to a specific fraction of the myeloid cell compartment.

### Formation of granuloma is partially independent on adaptive immunity

**Figure 6:**
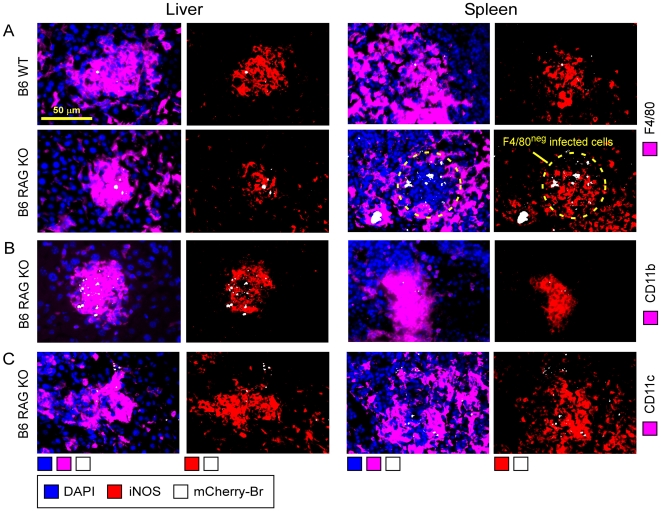
Phenotypical analysis of granuloma in the liver and spleen of wild type and RAG-deficient infected mice. Localization by immunohistofluorescence of (**A**) F4/80, (**B**) CD11b, (**C**) CD11c and iNOS expressing cells in liver and spleen from 120 h mCherry-Br infected wild-type and RAG^−/−^ C57BL/6. Mice were injected i.p. with 10^8^ CFU of mCherry-Br. Images represent a single granuloma. Panels are color-coded with the text for the antigen or mCherry-Br examined. Scale bar = 50 µm, as indicated. Data are representative of at least 3 independent experiments.

As granulomas seem to be the main organized cellular structures containing *Brucella* in the spleen and the liver, we tried to define the role of T and B lymphocytes in their formation. We infected RAG deficient C57BL/6 mice with 10^8^ CFU of mCherry-Br. RAG^−/−^ mice displayed higher CFU counts in the spleen, but not in the liver, at 120 h p.i. (Figure S11). At the same time, microscopy analysis of tissue sections showed that absence of T and B lymphocytes did not impair F4/80^+^ CD11b^+^ CD11c^+^ iNOS^+^ granuloma formation in either tissues (Figure 6). Flow cytometry analyses confirmed that recruitment of iNOS-DC was not compromised in the spleen of RAG^−/−^ infected mice (Figure S12). However, in RAG^−/−^ infected spleens, granulomas seemed less organized and less dense. In addition, higher numbers of iNOS^+^ cells and infected cells were observed outside of granulomas and frequency of F4/80^−^ infected cells was higher when compared to wild type mice (Figure 6). These latter observations could be correlated with the presence of Ly-6G^+^ infected cells in the spleen of RAG^−/−^ mice (data not shown).

### MyD88, IL-12 and IFNγ are required for granuloma formation

In order to gain insight into immune mechanisms controlling bacterial growth in the spleen, we used a genetic approach to investigate the impact of MyD88, IL-12p35 and IFN-γ deficiencies on the phenotype of infected cells with a particular focus on the ability of the immune response to establish granulomas. These molecules have been described as key elements of Th1-driven immune response controlling *Brucella* growth *in vivo* [10],[14], [28],[12]. In agreement with previous published results, all deficient mice displayed higher CFU counts in spleen and liver at 120 h p.i. (Figure S11). As expected, frequencies of IFN-γ^+^ cells and iNOS-DC in infected spleen were drastically reduced in all Th1 deficient mice when compared to infected wild type mice (data not shown and Figure S12).

**Figure 7:**
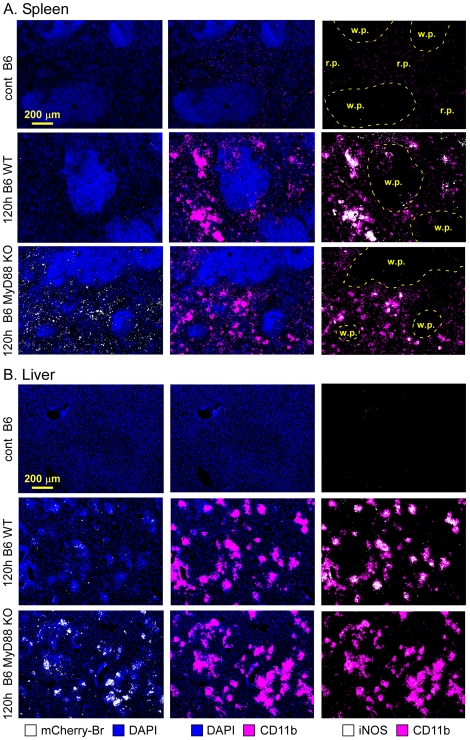
CD11b^+^ granulomas in the spleen and liver of wild type and MyD88-deficient infected mice. Mice were injected i.p. with PBS (control) or 10^8^ CFU of mCherry-Br. The figure presents the localization by immunohistofluorescence of CD11b^hi^ and iNOS expressing cells in spleen (**A**) and liver (**B**) from control and 120 h mCherry-Br infected wild-type and MyD88^−/−^ C57BL/6. Panels are color-coded with the text for the antigen or mCherry-Br examined. Scale bar = 200 µm, as indicated. r.p.: red pulp; w.p.: white pulp. Data are representative of at least 3 independent experiments.

**Figure 8:**
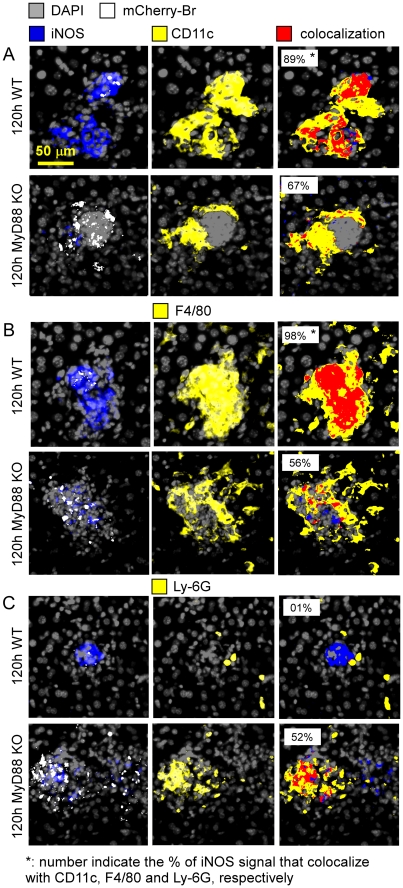
Phenotypical analysis of granuloma in the liver of wild type and MyD88-deficient infected mice. Colocalization by immunohistofluorescence of CD11c (**A**), F4/80 (**B**), Ly-6G (**C**) and iNOS expressing cells with mCherry-Br signal in liver from 120 h mCherry-Br infected wild-type and MyD88^−/−^ C57BL/6. Mice were injected i.p. with 10^8^ CFU of mCherry-Br. Panels are color-coded with the text for the antigen or mCherry-Br examined. Scale bar = 50 µm, as indicated. Data are representative of at least 3 independent experiments.

Immunofluorescence analysis of liver and spleen sections from 120 h-infected mice showed that all three deficient mouse strains displayed dense aggregates of CD11b^+^ cells surrounding infected cells and resembling granulomas (Figure 7 for spleen and liver from wild type and MyD88^−/−^ mice and data not shown for IL-12^−/−^ and IFN-γ^−/−^ mice). Whereas iNOS staining was strongly reduced in all three deficient mouse strains (Figure 7 and data not shown), CD90.2^+^ T cells recruitment around infected cells was normal (data not shown). Moreover, granuloma-like structures in all deficient mouse strains frequently contained higher numbers of infected Ly-6G^+^ cells but reduced density of F4/80^+^ and CD11c^+^ cells (Figure 8 for liver granulomas and data not shown for spleen granulomas). These “unconventional” granulomas, characterized by an inverted ratio of granulocytes versus monocytes/DC, failed to control bacterial growth as demonstrated by an increased mCherry-Br signal in deficient mice, with higher numbers of infected cells outside of granuloma like structures. These “unconventional” granulomas were also detected in infected wild type mice but at a low frequency.

### Major cellular reservoir for *Brucella* in spleens of chronically infected susceptible mice localizes in white pulp and expresses MOMA-1 or CD11c and CD205 markers

**Figure 9:**
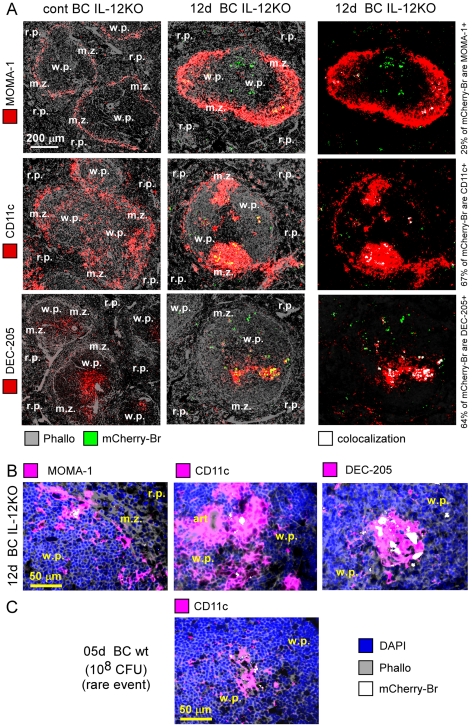
*B. melitenis* infects CD11c^+^ CD205^+^ or MOMA-1^+^ cells in spleens of chronically infected Il-12-deficient BALB/c mice. Wild-type and Il-12p40^−/−^ BALB/c mice were injected i.p. with PBS or mCherry-Br, as indicated. Mice were sacrificed 5 or 12 days p.i. and spleens were collected and examined by immunohistofluorescence. **A** & **B**, Colocalisation of mCherry-Br with MOMA-1, CD11c, CD205 expressing cells in the spleen of IL-12^−/−^ BALB/c mice infected with 10^6^ CFU 12 days p.i.. **C**, Higher magnification view of CD11c-expressing cells and mCherry-Br in w.p. of wild type BALB/c mice infected with 10^8^ CFU 5 days p.i.. Panels are color-coded with the text for the antigen or mCherry-Br examined as well as the colocalization. Scale bar = 200 and 50 µm, as indicated. r.p.: red pulp; w.p.: white pulp, m.z.: marginal zone. Data are representative of at least 3 independent experiments.

One of our initial objectives was to characterize the phenotype of *Brucella* reservoir cell types in chronically infected mice. Unfortunately, the detection threshold of mCherry-Br signal *in situ* required approximately 10^6^ CFU/g of tissue (Figure S3.A and B). Wild type mice initially infected with 10^8^ bacteria displayed CFU counts in spleen and liver inferior to this threshold at 12 days p.i. and MyD88^−/−^, IL-12^−/−^ or IFN-γ^−/−^ mice showed high mortality rate at the same time point. Thus, in order to increase the lifespan of genetically deficient mice, we tested an inoculum dose of 10^6^ CFU and compared bacterial loads in spleen and liver at 5, 12 and 30 days p.i. (Figure S13). Only IL-12p40^−/−^ BALB/c mice displayed CFU counts superior to the detection threshold in spleens at 12 and 30 days p.i. Interestingly, microscopy analysis of tissue sections from spleens from infected IL-12^−/−^ BALB/c mice (Figure S14.B) showed that mCherry-Br signal, initially located in r.p. at 12 h p.i., progressively relocated in w.p. at 5, 12 and 30 days p.i. This preferential localization in w.p. was also observed in infected IL-12^−/−^ C57BL/6 at 5 days p.i. (Figure S14.C), demonstrating that the BALB/c background is dispensable to observe this phenomenon. Surprisingly, the phenotype of *Brucella*-containing cells located in w.p. of 12 days-infected IL-12^−/−^ BALB/c mice was strikingly distinct from r.p. infected cells previously described in wild type mice (Figure 9.A and 9.B). Indeed, these cells were highly infected (>20 bacteria/cell) and harbored two distinct cell surface marker combinations. When localized in close proximity of m.z. area, the infected cells expressed MOMA-1, a specific marker of metallophilic marginal zone macrophages, whereas when located deeply within the w.p., they expressed CD11c and DEC205/CD205 (Figure 9.B), both DC-specific molecules. Both types of cells were negative for iNOS, F4/80, CD11b, CD86, CD90.2 and B220 (data not shown) and a fraction of them expressed weakly MHC-II. Although the bacterial load was reduced, the phenotype and location of IL-12^−/−^ infected cells was similar 30 days p.i., (data not shown). Interestingly, highly infected CD11c^+^ cells located in the w.p. of wild-type BALB/c mice could also be observed 5 days p.i. but at a very low frequency (Figure 9.C and data not shown) suggesting that those cells are not limited to Il-12^−/−^ BALB/c mice.

Given that these heavily infected w.p. cells were not found associated with CD11b^+^ F4/80^+^ iNOS^+^ effector cells, we conclude that they may constitute an important reservoir for *B. melitensis* under permissive conditions such as absence of Th1 protective response.

## Discussion

Bacteria of the genus *Brucella*, have been long studied as a relevant experimental model to analyze chronic infections of animals and humans due to the great impact they have on both husbandry practice and human health worldwide [29]. Their virulence and the chronicity of the ensuing disease rely on their ability to modulate both innate and adaptive immune responses and the physiology of the host's cells in which they reside, survive and multiply (reviewed in [30]).

The mouse is considered as a useful animal model to investigate the pathogenesis of brucellosis, to identify specific virulence factors of *Brucella* spp and to characterize the host immune response [29]. If classical studies [31], [32], and more recently the use of genetically deficient mice, have been useful to uncover the great avenues of protective immune responses against *Brucella* infection [33], this approach appears insufficient to dissect immune effector mechanisms elaborated by the host to fight *B. melitensis*. Like *Mycobacterium tuberculosis* [34], [35],[36], and in contrast to other intracellular bacteria such as *Listeria monocytogenes* [37] or *Legionella pneumophila* [38], *B. melitensis* does not irreversibly affect the health status of the majority of mice genetically deficient for key element of Th1 protective immune response (such as MyD88 [10],[39], IL-12p40 [11], IFN-γ [12] and iNOS [11]) during the first four weeks of infection. These observations suggest that immune effector mechanisms against *Brucella* are multiple and redundant, and necessitate new approaches to be further characterized. This is the perspective from which we decided to use *in situ* microscopy techniques for studying the local immune response developed around *B. melitensis* infected cells. Up to now, only gross morphometric and histopathologic analyses have been conducted [40]–[42] on spleen and liver, that are important sites for colonization and replication of *Brucella* in the mouse model [29]. Both organs develop the so called “histiocytic infiltrates and microgranulomas” [41], [42]. Of importance, *Brucella* infection in mice results in lesions mimicking those described in chronic infections in humans which may also develop splenomegaly and hepatomegaly [17], [43]. To our knowledge, our study and the recent work from Archambaud, C *et al.* [44] are the first *in vivo* investigations characterizing the *in vivo* phenotype of *Brucella*-infected cells. Archambaud, C *et al.* have focused their study on *Brucella abortus*-infected pulmonary tissues following intranasal inoculation. In contrast, we used an i.p. systemic model of infection in order to analyse the phenotype of *Brucella melitensis*-infected cells in spleen and liver. The i.p. route of infection is generally used to establish a persistent infection in the mouse because it leads to a rapid systemic distribution of *Brucella* sp. and to high bacterial loads in both spleen and liver [33], [45].

*Brucella* has been initially described as an intracellular bacteria able to replicate in professional phagocytes such as macrophages [46], DC [47] and granulocytes as well as non-professional phagocytes [48], including epithelial, fibroblastic and trophoblastic cells, in the context of cell line cultures. However, the identity of *Brucella* reservoir cell types during the course of infection *in vivo* is largely uncharacterized. If macrophages [40] and trophoblastic cells [49] have been clearly associated with *Brucella* infection in the natural hosts, little has been demonstrated regarding the contribution of other potential cellular niches for *Brucella in vivo*.

In this study, using a *Brucella* strain stably expressing mCherry, we demonstrated for the first time that these bacteria present a very restrictive cellular tropism as the majority of infected cells in the spleen and the liver of resistant C57BL/6 wild type mice belongs to a specific fraction of the myeloid lineage. Granulocytes, B cells, T cells, fibroblasts and hepatocytes were never found significantly infected. This result may explain the frequent discrepancies between *in vitro* and *in vivo* attenuation of various *Brucella* mutants. In the physiological cell reservoir and in the complex microenvironment of the host, some of the “virulence factors” identified *in vitro* may have little or no relevance. A striking example is the mutation affecting the gene *virB* which encodes the type IV secretion system of *Brucella*. The *virB* mutant is unable to grow within host cells *in vitro* [22] though it can replicate at a similar level as the wild type strain during the first 5 days of infection *in vivo* [23]. In the present study, *virB* mutant and wild type bacteria were localized within the same cells in infected spleen at all time-points analyzed during the first three days of infection. This argues in favor of the intracellular localization of *virB* mutant *in vivo* and strongly suggests that the type IV secretion system of *Brucella* is tightly regulated during the infectious process. Since we showed that the nature of the infected cells varied in a time-dependent manner during the first days of infection, it is tempting to speculate that the type IV secretion system depends on the nature of the infected cells and/or its activation status.

Our study also brought to light the complexity and the dynamic of the cellular environment of the pathogen during the course of infection. In the spleen, m.z. (MOMA-1^+^) and r.p. (F4/80^+^) macrophages are the first infected cells, followed by DCs (CD11c^+^) located in m.z. and r.p.. We hypothesized that infection-mediated inflammation is responsible of subsequent recruitment of CD11b^+^ Ly-6G^−^ monocytes. Our data suggest that these cells are rapidly infected and progressively mature to form complex granulomas in r.p. composed of a mix of activated mature monocytes (CD11b^+^ CD11c^−^ F4/80^+^ MHC-II^+^) and inflammatory DC (CD11b^+^ CD11c^+^ F4/80^+^ MHC-II^+^) and surrounded by T cells (CD90.2^+^) and granulocytes (CD11b^+^ Ly-6G^+^). This scenario yields major differences when compared with the observations by Archambaud et al. [44] in the context of intra-nasal infection with *Brucella abortus*. Although macrophages are the first infected cells in spleen, liver, as shown in our study, and lungs [44], DC infection and granulomas formation are not observed in lungs but only in draining lymph node [44]. These discrepancies could be due to differences in dose of infection, route of inoculation and bacterial strain used in both studies.

It has been frequently reported that BALB/c mice display higher bacterial loads per organ when compared to C57BL/6 mice during *Brucella* infection [10], [25]. The reasons of this susceptibility are unclear. In our experimental model, spleen and liver infected cells displayed a closely similar phenotype and localization. However, higher CFU counts observed in infected BALB/c mice was related to increase colonization of w.p. cells. These latter displayed high number of bacteria and expressed MOMA-1 or CD11c markers but were negative for CD11b, F4/80, Ly-6G and CD90.2,

Transient infection of CD11c^+^ cells during the early stages of infection in the spleen leads us to characterize the impact of *Brucella* infection on the maturation of conventional DC. Flow cytometry and *in situ* staining showed that spleen infection with *B. melitensis* induced the massive migration of DC in the T cell area of w.p. as well as their maturation during the first 24 h of infection, followed later, by their gradual disappearance. The relationship between *Brucella* and DCs seems complex and necessitates further experiments to be elucidated. However, our results demonstrate clearly that the course of *Brucella* infection is associated to DCs activation in spleen. This maturation is dependent of MyD88 adapter molecule and does not require infection as heat killed *Brucella* induce a similar phenomenon. Importantly, microscopy analysis indicated that none of CD11c^+^ mature cells migrating to T cell area were infected by *B. melitensis* (data not shown), suggesting that infection of DCs could impair their migration and maybe their maturation. Again, these results are in agreement with the stealthy strategy [30] attributed to *Brucella* and with *in vitro* studies [50],[51] demonstrating the ability of *Brucella* to regulate DC maturation.

Using genetically deficient mice, we partially clarified some requirements for granuloma formation during *Brucella* infection. Development of a Th1 response seems a critical step as MyD88^−/−^, IL-12p35^−/−^ and IFN-γ^−/−^ mice displayed iNOS^−^ altered granulomas, strongly enriched in granulocytes. In contrast, the analysis of RAG^−/−^ infected mice demonstrated that granuloma formation at 5 days p.i. is, for a large part, independent of lymphocytes. We hypothesized, in accordance with the granuloma formation model proposed for *Listeria monocytogenes* infection [16], that IFN-γ is needed for maturation of monocytes in iNOS-DC and building of fully functional granuloma. As previously described [10], IFN-γ was produced by Natural Killer (NK) cells and T lymphocytes during *Brucella* infection. In RAG^−/−^ mice, iNOS-producing DC (iNOS-DC) were detectable by flow cytometry and microscopy analysis suggesting that absence of IFN-γ-producing T lymphocyte might be compensated by IFN-γ-producing NK cells. This was confirmed by the absence of iNOS-DC in spleens of RAGγc^−/−^ (deficient for natural killer cells) infected mice (data not shown).

The presence of phenotypically similar granulomas in the infected spleen and liver support the hypothesis that granulomas constitute the “spearhead” of the immune response against *Brucella*. Correlation between high susceptibility of MyD88^−/−^, IL-12^−/−^ and IFN-γ^−/−^ mice and strongly altered granuloma structures in these mice suggested a causal link between the presence of granulomas and *Brucella* growth control. However, the precise role of granuloma during *Brucella* infection remains to be clarified.

A prominent characteristic of *Brucella* is its capacity to persist in natural host for life (reviewed in [30]). This property is thought to be related to its capacity to (i) locate intracellularly and avoid the fusion of *Brucella*-containing vacuoles with host cell lysosomes (ii) limit or modulate the activation of innate and adaptive immune response (mainly due to poorly recognized PAMPs of its cell envelope and poorly characterized secreted effectors), (iii) prolong the lifespan of infected cells. The previously unappreciated restricted specificity of *Brucella* cell tropism that we observed *in vivo* allows us to hypothesize that the ability of *Brucella* to persist could also depend of its capacity to infect cell subsets particularly adapted to sustain its growth and persistence. In absence of Th1 response, granuloma formation was altered and the bacterial burden was significantly higher in spleens of IL-12p40^−/−^ BALB/c mice, making possible microscopy observation *in situ* and characterization of infected cells during chronic phase of infection (12 and 30 days p.i.). Our analyses revealed that the main infected spleen cells at these time-points are located in the w.p. and express the following cell surface phenotype: CD11b^−^ CD11c^+^ CD90^−^ CD205^+^ F4/80^−^ Ly-6G^−^ MHC-II^low^ B220^−^ iNOS^−^. High expression of CD11c and CD205 suggested that these cells are a particular subset of DC. This phenotype is also partially reminiscent of foamy macrophages that express high levels of CD11c, CD205 and low level of MHC-II [52] and constitute nutrient-rich reservoirs in lung granulomas of *M. tuberculosis*-infected mice [53]. Interestingly, these cells were also detected, albeit with a low frequency, five days post-infection in the w.p. of wild type BALB/c mice infected with 10^8^ CFU. Previously, we demonstrated the reduced ability of BALB/c mice to mount Th1 [10] response in the context of *Brucella melitensis* infection. Thus, permissive environments, such as the absence of Th1 response, may drive the differentiation of macrophages toward a long-lived and anti-inflammatory phenotype allowing bacterial persistence. Identification of these cells as potential reservoir for *Brucella* in chronically infected mice could help to ameliorate therapeutic treatment of brucellosis.

In conclusion, this work dissected for the first time the nature of the effectors mechanisms developed *in vivo* by the immune system after *B. melitensis* systemic inoculation and described the phenotypic characteristics of infected cells during the initial and chronic steps of the infectious process. These results could help develop new strategies to control *Brucella* infection.

## Materials and Methods

### Ethics statement

The animal handling and procedures of this study were in accordance with the current European legislation (directive 86/609/EEC) and in agreement with the corresponding Belgian law “*Arrêté royal relatif à la protection des animaux d'expérience du 6 avril 2010 publié le 14 mai 2010*”. The complete protocol was reviewed and approved by the Animal Welfare Committee of the Facultés Universitaires Notre-Dame de la Paix (FUNDP, Belgium)(Permit Number: 05-558).

### Mice and reagents

Genetically deficient mice in C57BL/6 background: MyD88^−/−^ [54] were obtained from Dr. S. Akira (Osaka University, Japan). TRIF^−/−^ mice [55] were a kind gift from Dr. B. Beutler (The Scripps Research Institute, CA), IL-12p35^−/−^ mice [56] from Dr. B. Ryffel (University of Orleans, France), IFN-γ^−/−^ mice [57] from Dr. S. Magez (Vrije Universiteit Brussel, Belgium), RAG1^−/−^ mice [58] from Dr. S. Goriely (Université Libre de Bruxelles, Belgium), RAGγc^−/−^ mice from Dr. Michel Y. Braun (Université Libre de Bruxelles, Belgium). IL-12p40^−/−^ BALB/c mice were obtained from Dr. V. Flamand (Université Libre de Bruxelles, Belgium). Wild type C57BL/6 mice and BALB/c mice purchased from Harlan (Bicester, UK) were used as control. All mice used in this study were bred in the animal facility of campus Gosselies from the Free University of Brussels (ULB, Belgium).

*B. melitensis* strain 16M (Biotype1, ATCC 23456) was isolated from an infected goat and grown in biosafety level III laboratory facility. Overnight culture grown with shaking at 37°C in 2YT media (Luria-Bertani broth with double quantity of yeast extract) to stationary phase was washed twice in PBS (3500×g, 10 min.) before use in mice inoculation.

### Construction of a mCherry-producing *B. melitensis* strain

The mCherry protein, a previously described rapidly maturing variant of the red fluorescent protein DsRed [18], was cloned into the suicide vector pKSoriT-*bla*-*kan* downstream a strong *Brucella* spp. promoter [19], [20], *nouvelle ref* Köhler S, Infect Immun. 1999) previously used to express constitutively the GFP. This promoter was called P*sojA* and described as controlling the expression of the protein translocase SecE (Köhler S.; personal communication).

The vector was constructed as follows: the mCherry coding sequence was amplified by PCR from pRSET-B-mCherry [18] with the mCherry-up and -down primers and ligated into pGemT-Easy (Promega) to generate pGEM-T-mCherry. The mCherry fragment was then excised from pGEM-T-mCherry by HindIII/XbaI double restriction and subsequently cloned into HindIII/XbaI-cut pKSoriT-*bla* (pBluescript II KS vector from Stratagene in which RP4 oriT was inserted in order to make this vector mobilizable [59]) to generate pKSoriT-*bla*-*mCherry*. Meanwhile, a 500 bp fragment upstream the coding sequence of secE was amplified from the *B. melitensis* genome by PCR with the P*sojA*-up and Ps*ojA*-down primers and ligated into pGemT-Easy (Promega) to generate pGEM-T-*SojA*. The P*sojA* fragment was then excised from pGEM-T-P*sojA* by NotI/XbaI double restriction and subsequently inserted into NotI/XbaI-cut pKSoriT-*bla*-*mCherry* to generate pKSoriT-*bla*-P*sojA*-*mCherry*. Finally, the aphA4 cassette (a promoterless kanamycin resistance gene [60] was excised from pUC4aphA4 with SalI, and subsequently cloned into the XhoI site of pKSoriT-*bla*-P*sojA*-*mCherry* to generate plasmid pKSoriT-*bla*-*kan*-P*sojA*-*mCherry*. This final construct was transformed into *E. coli* strain S17-1, and introduced into *B. melitensis* 16M NalR strain by conjugation. Clones that were kanamycin resistant and fluorescent were further checked by PCR, confirming the insertion of the plasmid at the targeted chromosomal P*sojA* promoter. Primers used in this study (Sequence (5′-3′)): mCherry-up (XbaI) tctagaatggtgagcaagggcgag, mCherry-down (HindIII) aagcttttacttgtacagctcgtcca, PsojA-up (NotI) gcggccgccttgactatggatgcccgtt, PsojA-down (XbaI) tctagactctgtctgatcaggcacaa.

Similar protocol has been used to construct mCherry-expressing ΔVirB *B. melitenis*. Construction of ΔVirB mutant has been previously described [61].

### Mouse infection

Mice were injected intra-peritoneally (i.p.) with indicated dose of *B. melitensis* in 500 µl of PBS. Control animals were injected with the same volume of PBS. Infectious doses were validated by plating serial dilutions of inoculums. At selected time intervals, mice were sacrificed by cervical dislocation. Immediately after being killed, spleen and liver were collected for bacterial count and flow cytometry and microscopy analyses.

### Bacterial count

For bacterial count, spleens and livers were recovered in PBS/0.1% X-100 triton (Sigma). We performed successive serial dilutions in PBS to get the most accurate bacterial count and we plated them onto 2YT media plates. CFU were counted after 3 days of culture at 37°C.

### Cytofluorometry analysis

Spleen were harvested, cut in very small pieces and incubated with a cocktail of DNAse I fraction IX (Sigma-Aldrich Chimie SARL, Lyon, France) (100 µg/ml) and 1.6 mg/ml of collagenase (400 Mandl U/ml) at 37°C for 30 min. After washing, spleen cells were filtered and incubated in saturating doses of purified 2.4G2 (anti-mouse Fc receptor, ATCC) in 200 µl PBS 0.5% BSA 0.02% NaN3 (FACS buffer) for 10 minutes on ice to prevent antibody binding to Fc receptor. 3–5×10^6^ cells were stained on ice with various fluorescent mAbs combinations in FACS buffer and further collected on a FACScalibur cytofluorometer (Becton Dickinson, BD). We purchased the following mAbs from BD Biosciences: Fluoresceine (FITC)-coupled 145-2C11 (anti-CD3ε), 53-8.7 (anti-CD8ε), M1/70 (anti-CD11b), GL-1 (anti-CD86), Phycoerythrin (PE)-coupled HL3 (anti-CD11c), RM4-5 (anti-CD4). Allophycocyanin (APC)-coupled M5/114.15.2 (anti-IA/IE). The cells were analyzed on a FACScalibur cytofluorometer. Cells were gated according to size and scatter to eliminate dead cells and debris from analysis.

### Intracellular cytokine staining

Spleen cells were treated as previously described [62]. Spleen cells were incubated for 4 h in RPMI 1640 5% FCS with 1 µl/ml Golgi Plug (BD Pharmingen) at 37°C, 5% CO2. The cells were washed with FACS buffer and stained for cell surface markers before fixation in PBS/1% PFA for 15–20 min on ice. These cells were then permeabilized for 30 min using a saponin-based buffer (1× Perm/Wash, BD Pharmingen in FACS buffer) and stained with one or a combination of the following intracellular mAbs: allophycocyanin-coupled XMG1.2 (anti-IFN-γ; BD Biosciences), purified M-19 (rabbit polyclonal igG anti-NOS2; Santa Cruz Biotechnology) stained with Alexa Fluor 647 goat anti-rabbit (Molecular Probes). After final fixation in PBS/1% PFA, cells were analyzed on a FACScalibur cytofluorometer. No signal was detectable with control isotypes.

### Immunofluorescence microscopy

Spleens and livers were fixed for 6 h at 4°C in 2% paraformaldehyde (pH 7.4), washed in PBS, incubated overnight at 4°C in a 20% PBS-sucrose solution under agitation, and washed again in PBS. Tissues were embedded in the Tissue-Tek OCT compound (Sakura), frozen, in liquid nitrogen, and cryostat sections (5 µm) were prepared. Tissues sections were rehydrated in PBS, then incubated successively in a PBS solution containing 1% blocking reagent (Boeringer) (PBS-BR 1%) and in PBS-BR 1% containing any of the following mAbs or reagents: DAPI nucleic acid stain, Alexa Fluor 350 or 488 phalloidin (Molecular Probes), purified 1A8 (anti-Ly-6G), or rabbit polyclonal antibodies anti-NOS2 (Calbiochem) (note that M-19 anti-NOS2; used for cytofluorometry analysis is not use for immunofluorescence microscopy), biotin-coupled HL3 (anti-CD11c, BD Biosciences), NLDC-145 (anti-DEC205/CD205, BMA Biomedical AG), MOMA-1 (anti Marginal Zone Macrophages, BMA Biomedicals), 53-2.1 (anti-CD90.2, BD Biosciences), RA3-6B2 (anti-CD45R/B220, BD Biosciences), Alexa Fluor 647-coupled BM8 (anti-F4/80, Abcam) M1/70 (anti-CD11b, BD Biosciences), M5/114.15.2 (anti-IA/IE, eBiosciences). Uncoupled 1A8 mAb and anti-NOS2 polyclonal antibodies were detected using biotin-coupled R67/1.30 (mouse anti-rat IgG2a, BD Biosciences) and Alexa Fluor 647-coupled goat anti-rabbit IgG (Molecular Probes) in PBS-BR 1%, respectively. Biotin-coupled mAbs were amplified using Alexa Fluor 350 or Alexa Fluor 647 Streptavidin (Molecular Probes) in PBS-BR 1%. Slides were mounted in Fluoro-Gel medium (Electron Microscopy Sciences, Hatfield, PA). Labeled tissues sections were visualized with an Axiovert M200 inverted microscope (Zeiss, Iena, Germany) equipped with high resolution monochrome camera (AxioCam HR, Zeiss). Images, 1384×1036 pixels (0.16 µm/pixel), were acquired sequentially for each fluorochrome with A-Plan 10×/0.25 N.A. and LD-Plan-NeoFluar 63×/0.75 N.A. dry objectives and recorded as eight bit grey levels *.zvi files. Colocalization between two stainings was analyzed using the AxioVision Colocalization module (Zeiss). Double positive pixels were rendered in white, gray or yellow as indicated in the Figures. Images were exported as TIFF files and figures prepared in Canvas 7 program.

For the estimation of the number of bacteria by cells or for the phenotype of infected cells, a minimum of 200 cells by condition were examined. These cells were counted in 6 mice minimum, in two independent experiments. When the number of bacteria by individual cell was too high to be determined, the number of bacteria was assumed to be of 20 or more (see Figure 1.E).

### Confocal analysis

Confocal analysis were performed with LSM510 NLO multiphoton confocal microscope fitted on an Axiovert M200 inverted microscope equipped with C-Apochromat 40×/1.2 N.A. water immersion objectives (Zeiss). Optical sections of 1 µm thick, 568×568 pixels (0.1 µm/pixel), were collected sequentially for each fluorochrome and recorded as eight bit grey levels *.lsm files.

### Statistical analysis

We have used a (Wilcoxon-) Mann-Whitney test provided by GraphPad Prism program to statistically analyze our results. Each group of deficient mice was compared to wild type mice. We also compared each group to each other and displayed the result when it is required. Values of p<0.05 were considered to represent a significant difference. *, **, *** denote p<0.05, p<0.01, p<0.001, respectively.

## Supporting Information

Figure S1 **Characterization of the mCherry-expressing fluorescent strain of** ***B. melitensis*** **16M (mCherry-Br).** **A**, Flow cytometry analysis of *B. melitensis* 16M and mCherry-Br. **B**, Infection of hamster fibroblastic cells BHK with *B. melitensis* 16M and mCherry-Br. The LPS of both bacteria is labelled in green and the mCherry-Br strain appears in red. **C**, Virulence curve of *B. melitensis* 16M and mCherry-Br. Groups of six mice were inoculated i.p. with 4×10^4^ CFU of both *Brucella* strains. At the indicated times the CFU per spleen were calculated. Data are representative of at least 3 independent experiments.
(TIF)

Figure S2 **Dynamic of dendritic cell, neutrophil and macrophage populations in spleen following low and high doses of bacteria.** Wild-type C57BL/6 mice (5 per groups) were inoculated i.p. with PBS, 4×10^4^, 10^7^ CFU or 10^8^ CFU of mCherry-Br, as indicated. At selected time, mice were sacrificed, spleens were collected and total spleen cells from each individual mouse were counted by Thoma cell. Cells were analyzed by flow cytometry, gated according to size and scatter to exclude dead cells and debris from analysis and then analyzed for CD11b, CD11c, F4/80, Ly-6C and Ly-6G expression, in order to obtain the frequencies of dendritic cell, neutrophil and macrophage subsets, as indicated at the top of each graph. The total number of each population by spleen was calculated and the data represent the median +/− SD of 5 mice. Data are representative of 2 independent experiments.
(TIF)

Figure S3 **Time course of mCherry-Br infection and number of infected spleen cells by surface unit.** **A**, Wild-type C57BL/6 and BALB/c mice (6 per groups) were inoculated i.p. with 10^8^ CFU of mCherry-Br. At the indicated times, CFU per spleen were calculated. These results are representative of three independent experiments. **B**, Graphic representation of the number of splenic infected cells by surface unit of spleen section at 24 h and 120 h p.i. A unit surface is defined arbitrary as an area of 0,037 mm^2^, corresponding to the tissue surface examined with 63× objective. The bars are the mean ± SD from at least 3 spleen sections per spleen from 5 mice.
(TIF)

Figure S4 **Co-localization of** ***B. melitensis*** **and** ***Brucella*** **antigens** ***in vivo*** **.** Colocalization analysis of F4/80 expressing cells, *Brucella* antigens and mCherry-Br before and 6 h after mCherry-Br infection in wild-type C57BL/6 mice. Mice were injected i.p. with PBS or 10^8^ CFU of mCherry-Br. Numbers indicate the percentage of colocalizing cells in the upper panel. The inset areas in the middle-right panels are shown in the bottom panels. Panels are color-coded with the text for the antigen or mCherry-Br examined. Scale bar = 200 and 50 µm, as indicated. Data are representative of at least 3 independent experiments. r.p.: red pulp; w.p.: white pulp; m.z.: marginal zone.
(TIF)

Figure S5 ***In vivo*** **intracellular localization of** ***Brucella*** **determined by** ***confocal*** **microscopy.** Wild-type C57BL/6 mice were injected i.p. with PBS or 10^8^ CFU of mCherry-Br. Mice were sacrificed at 1 day p.i. and the spleens were collected and examined by confocal microscopy. **A**, Representative confocal images of spleen section infected with mCherry-Br and labeled with antibodies against F4/80 (green) antigen. **B**, Deconvolution process of a spleen section infected with mCherry-Br and labeled with antibodies against F4/80 (green) antigen. **C**, Representative confocal images of spleen section infected with mCherry-expressing ΔvirB *B. melitensis* mutant and labeled with antibodies against F4/80 (green) antigen. m, micrometer.
(TIF)

Figure S6 ***B. melitensis*** **infects LY-6G^−^/CD11b^+^ during the course of infection.** Colocalization assay of CD11b, Ly-6G expressing cells and mCherry-Br, 72 h after infection in wild-type C57BL/6 mice. Mice were injected i.p. with PBS or 10^8^ CFU of mCherry-Br. Numbers indicate the percentage of colocalizing cells in the juxtaposing left panel. Panels are color-coded with the text for the antigen or mCherry-Br examined. Scale bar = 50 µm, as indicated. Data are representative of at least 3 independent experiments. r.p.: red pulp; w.p.: white pulp; m.z.: marginal zone.
(TIF)

Figure S7 **Granuloma formation in the spleen of mice infected by low and high doses of** ***B. melitensis*** **.** Wild-type C57BL/6 mice (5 per groups) were inoculated i.p. with PBS, 4×10^4^ or 10^8^ CFU of mCherry-Br, as indicated. At selected time, mice were sacrificed, spleens were collected and examined by immunohistofluorescence. Panels are color-coded with the text for the antigen examined (**A**), CD11b and (**B**) F4/80. Scale bar = 200 µm, as indicated. r.p.: red pulp; w.p.: white pulp; art.: central artery. Data are representative of 2 independent experiments.
(TIF)

Figure S8 ***B. melitensis*** **infection induces the DC migration in the spleen during the course of infection.** Positioning of DCs in the spleen of mice before and 12 or 24 h after mCherry-Br inoculation. Wild-type C57BL/6 mice were injected i.p. with PBS or 10^8^ CFU of mCherry-Br. Mice were sacrificed at selected times and spleens were collected and examined by immunohistofluorescence. Panels are color-coded with the text for the antigen examined. Scale bar = 200 µm, as indicated. r.p.: red pulp; w.p.: white pulp; art.: central artery. Data are representative of at least 3 independent experiments.
(TIF)

Figure S9 ***B. melitensis*** **infection induces the DC maturation.** Wild type, MyD88^−/−^ and TRIF^−/−^ C57BL/6 mice were injected i.p. with, 10^8^ CFU of live *B. melitenis* and various doses of heat killed (HK) *B. melitensis* and *Escherichia coli* O18:K1. Mice were sacrificed at selected times and spleens were collected and analyzed by flow cytometry. Cells were gated according to size and scatter to exclude dead cells and debris from analysis. **A**, Spleen cells from individual mice were first analyzed for Forward Size Scatter (FSC) and CD11c expression. CD11c^hi^ cells in each group were then analyzed for MHC-II and CD86 expression. Number indicates the percentage of positive cells per 10^6^ spleen cells acquired for the specified marker. **B, C**, Comparative analysis of CD86 level on CD11c^hi^ cells. The data are the median. Data are representative of at least 3 independent experiments.
(TIF)

Figure S10 **Phenotypical description of** ***Brucella*** **infected granuloma in the liver.** Wild-type C57BL/6 mice were injected i.p. with PBS or 10^8^ CFU of mCherry-Br. Mice were sacrificed at 5 days p.i. and livers were collected and examined by immunohistofluorescence. **A**, Immunofluorescence analysis of Ly-6G, CD90, CD11b, CD11c, F4/80, and MHC-II expressing cells and mCherry-Br. **B**, Percentage of iNOS^+^ cells that colocalizes with CD11b-, and CD11c-expressing cells. Images represent a single granuloma. Numbers in Figure B indicate the percentage of colocalizing cells in the upper panel. Panels are color-coded with the text for the antigen or mCherry-Br examined as well as the colocalization. Scale bar = 50 µm, as indicated. Data are representative of at least 3 independent experiments.
(TIF)

Figure S11 **Analysis of the bacterial load of the spleen and liver in wild type and genetically deficient mice.** Wild-type, IFN-γ^−/−^, IL-12p35^−/−^, MyD88^−/−^ and RAG^−/−^ C57BL/6 mice and wild-type and IL-12p40^−/−^ BALB/c mice (3–6 per group) were inoculated i.p. with 10^8^ CFU. 120 h p.i., CFU per g of spleen (**A**) and liver (**B**) were calculated. Data are pooled from 3 independent experiments displaying similar CFU mean for wild type mice.
(TIF)

Figure S12 **Flow cytometry analysis of iNOS-producing cells following** ***B. melitensis*** **infection.** Wild-type, IFN-γ^−/−^, IL-12p35^−/−^, MyD88^−/−^ and RAG^−/−^ C57BL/6 mice and wild-type and IL-12p40^−/−^ BALB/c mice (3–6 per group) were inoculated i.p. with 10^8^ CFU of *B. melitensis*. Mice were sacrificed 5 days post-infection and spleen were collected and analyzed by flow cytometry. Cells were gated according to size and scatter to exclude dead cells and debris from analysis. **A**, Total pooled spleen cells were first analyzed for Forward Size Scatter (FSC) and iNOS expression. iNOS^+^ cells in each group were then analyzed for CD11b and CD11c expression. Number indicates the percentage of cells in the selected quadrant. **B**, Number of CD11b^+^ CD11c^+^ iNOS^+^ cells per 5×10^5^ spleen cells acquired. Each data represents the value obtained from an individual spleen and the data are pooled from 4 independent experiments.
(TIF)

Figure S13 **Kinetic analysis of the bacterial load in the spleen and liver in wild type and genetically deficient mice.** Wild-type, MyD88^−/−^ and IL-12p35^−/−^ C57BL/6 mice and wild-type and IL-12p40^−/−^ BALB/c mice (4–8 per group) were inoculated i.p. with 10^6^ CFU. At 5, 12 and 30 days p.i., CFU per g of spleen (**A**) and liver (**B**) were calculated. Data are representative from 2 independent experiments.
(TIF)

Figure S14 **Localization of** ***B. melitensis*** **during the course of infection in wild type and Il-12-deficient BALB/c mice.** Wild-type and Il-12p40^−/−^ BALB/c and Il-12p35^−/−^ C57BL/6 mice were injected i.p. with PBS, 10^6^ CFU or 10^8^ CFU of mCherry-Br, as indicated. Mice were sacrificed at 12 h, 5 days, 12 days, and 30 days p.i., as indicated. Spleens were collected and examined by immunohistofluorescence. Positioning of mCherry-Br in the spleen of Wild-type (**A**), Il-12p40^−/−^ BALB/c (**B**) and Il-12p35^−/−^ C57BL/6 mice (**C**). Panels are color-coded with the text for the antigen or mCherry-Br examined. Scale bar = 200, as indicated. r.p.: red pulp; w.p.: white pulp. Data are representative of at least 3 independent experiments.
(TIF)
