# Supplementary figures and images for: In Situ Microscopy Analysis Reveals Local Innate Immune Response Developed around Brucella Infected Cells in Resistant and Susceptible Mice
Source: PLoS Pathog. 2012 Mar 29;8(3):e1002575. doi: 10.1371/journal.ppat.1002575 (PMC3315488; doi:10.1371/journal.ppat.1002575)

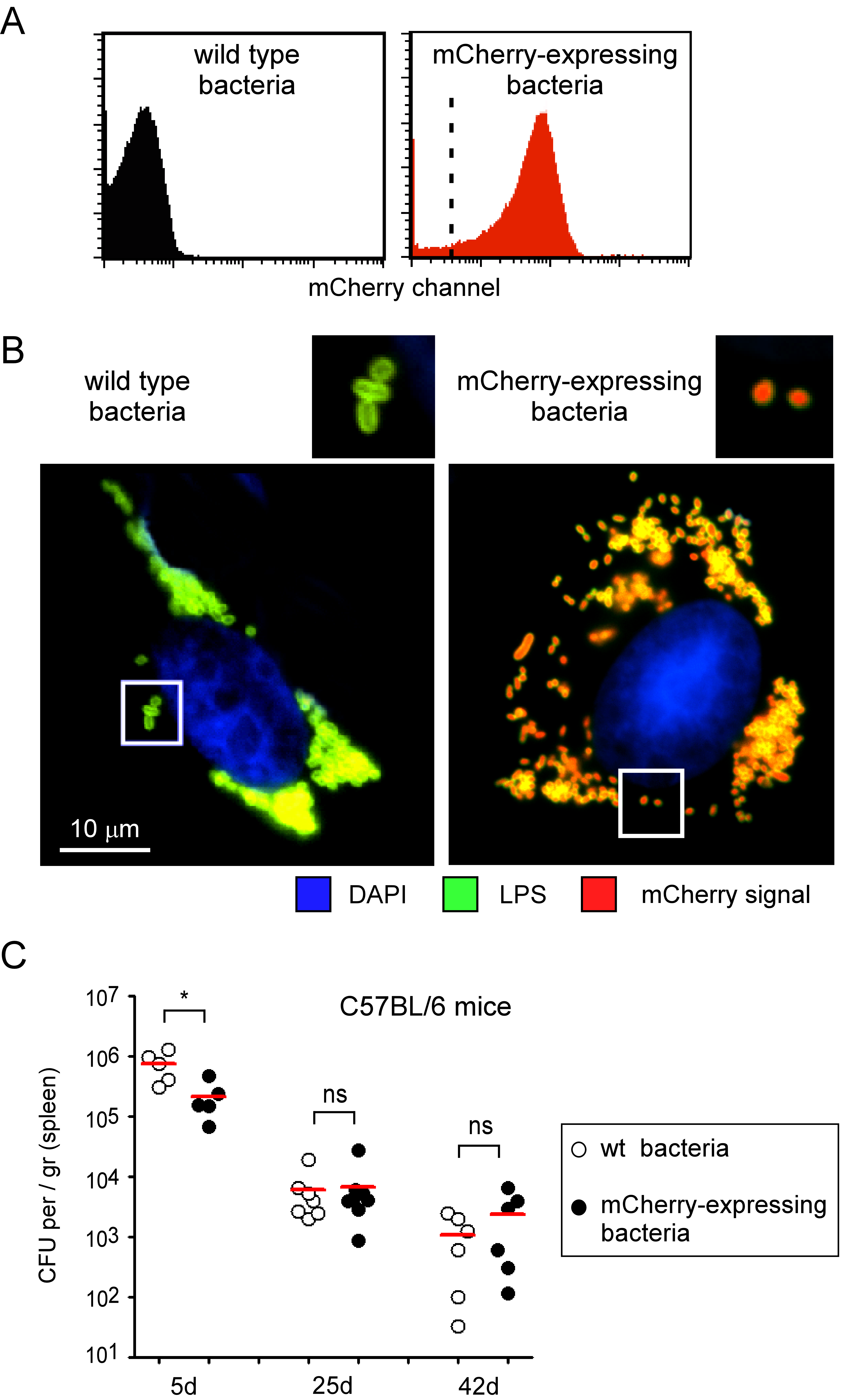

Supplement: Figure S1 — Characterization of the mCherry-expressing fluorescent strain of B. melitensis 16M (mCherry-Br). A, Flow cytometry analysis of B. melitensis 16M and mCherry-Br. B, Infection of hamster fibroblastic cells BHK with B. melitensis 16M and mCherry-Br. The LPS of both bacteria is labelled in green and the mCherry-Br strain appears in red. C, Virulence curve of B. melitensis 16M and mCherry-Br. Groups of six mice were inoculated i.p. with 4×10^4^ CFU of both Brucella strains. At the indicated times the CFU per spleen were calculated. Data are representative of at least 3 independent experiments. (TIF) [file ppat.1002575.s001.tif]

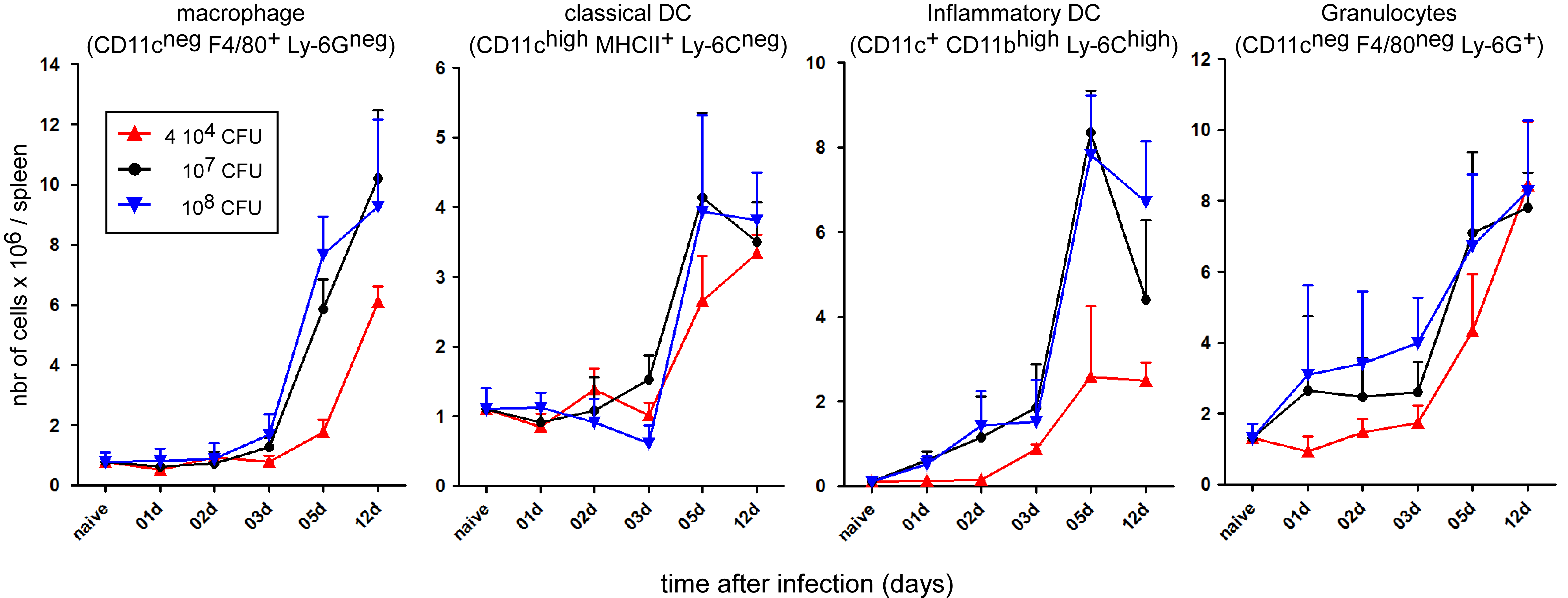

Supplement: Figure S2 — Dynamic of dendritic cell, neutrophil and macrophage populations in spleen following low and high doses of bacteria. Wild-type C57BL/6 mice (5 per groups) were inoculated i.p. with PBS, 4×10^4^, 107 CFU or 108 CFU of mCherry-Br, as indicated. At selected time, mice were sacrificed, spleens were collected and total spleen cells from each individual mouse were counted by Thoma cell. Cells were analyzed by flow cytometry, gated according to size and scatter to exclude dead cells and debris from analysis and then analyzed for CD11b, CD11c, F4/80, Ly-6C and Ly-6G expression, in order to obtain the frequencies of dendritic cell, neutrophil and macrophage subsets, as indicated at the top of each graph. The total number of each population by spleen was calculated and the data represent the median +/− SD of 5 mice. Data are representative of 2 independent experiments. (TIF) [file ppat.1002575.s002.tif]

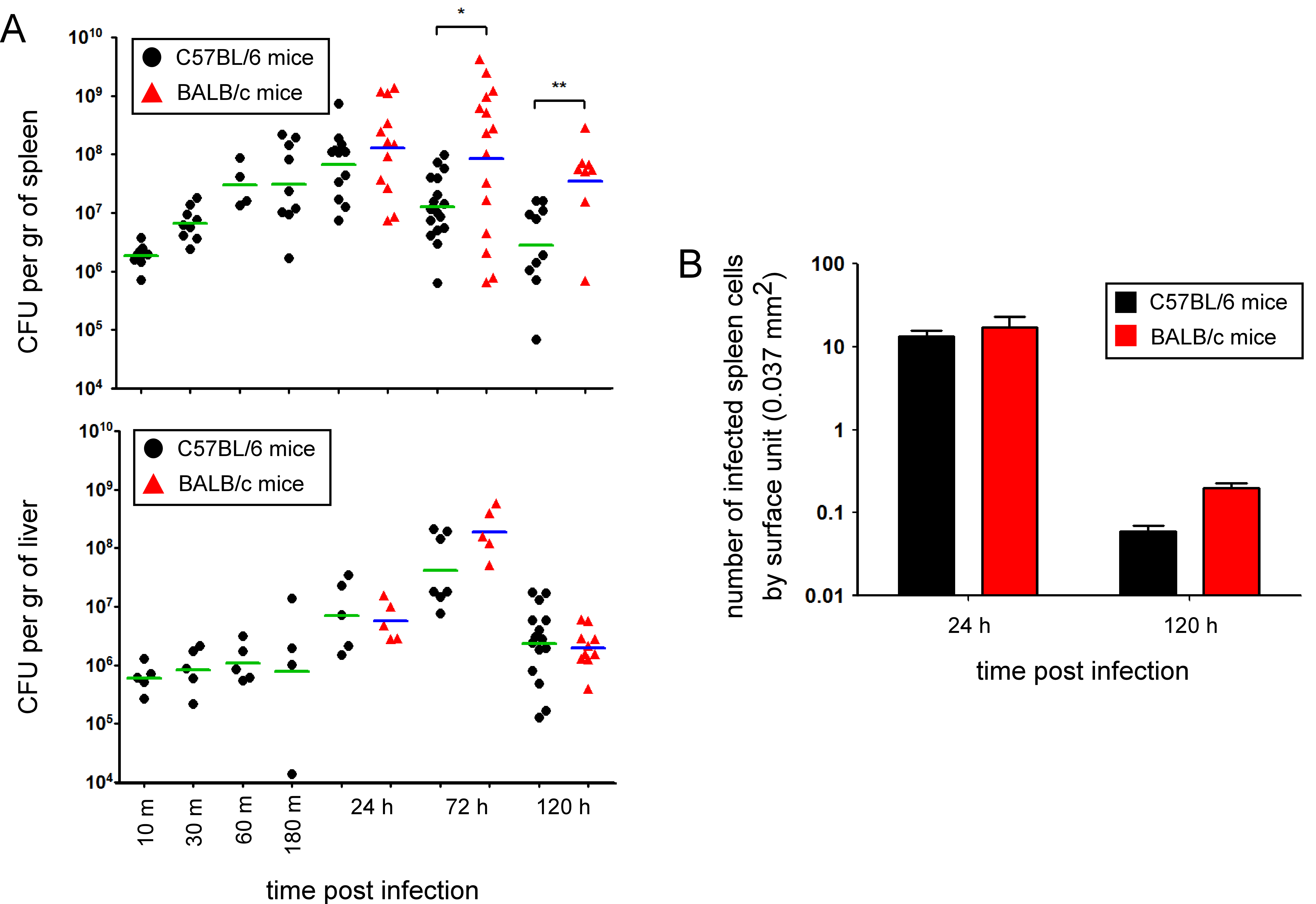

Supplement: Figure S3 — Time course of mCherry-Br infection and number of infected spleen cells by surface unit. A, Wild-type C57BL/6 and BALB/c mice (6 per groups) were inoculated i.p. with 108 CFU of mCherry-Br. At the indicated times, CFU per spleen were calculated. These results are representative of three independent experiments. B, Graphic representation of the number of splenic infected cells by surface unit of spleen section at 24 h and 120 h p.i. A unit surface is defined arbitrary as an area of 0,037 mm2, corresponding to the tissue surface examined with 63× objective. The bars are the mean ± SD from at least 3 spleen sections per spleen from 5 mice. (TIF) [file ppat.1002575.s003.tif]

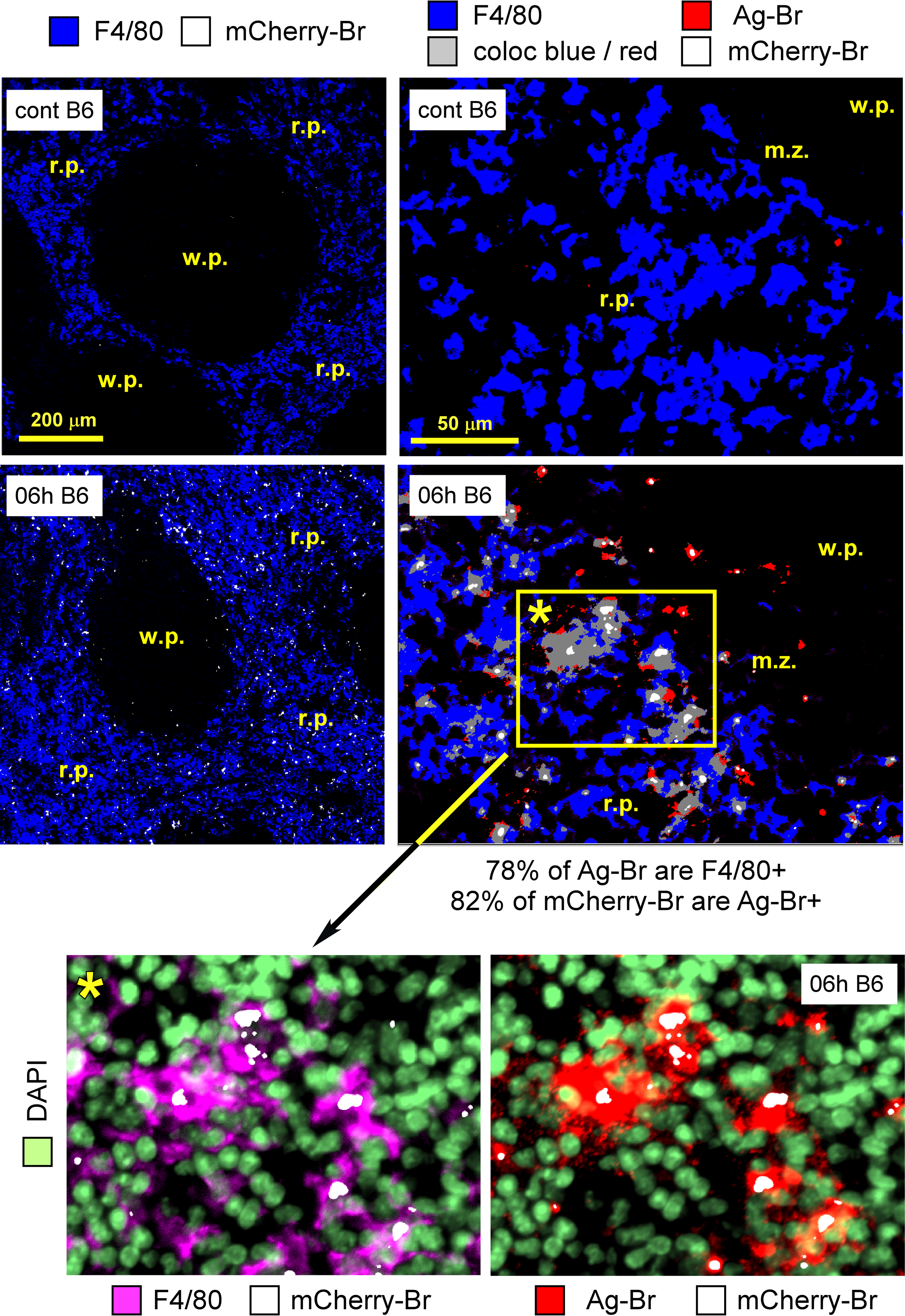

Supplement: Figure S4 — Co-localization of B. melitensis and Brucella antigens in vivo . Colocalization analysis of F4/80 expressing cells, Brucella antigens and mCherry-Br before and 6 h after mCherry-Br infection in wild-type C57BL/6 mice. Mice were injected i.p. with PBS or 108 CFU of mCherry-Br. Numbers indicate the percentage of colocalizing cells in the upper panel. The inset areas in the middle-right panels are shown in the bottom panels. Panels are color-coded with the text for the antigen or mCherry-Br examined. Scale bar = 200 and 50 µm, as indicated. Data are representative of at least 3 independent experiments. r.p.: red pulp; w.p.: white pulp; m.z.: marginal zone. (TIF) [file ppat.1002575.s004.tif]

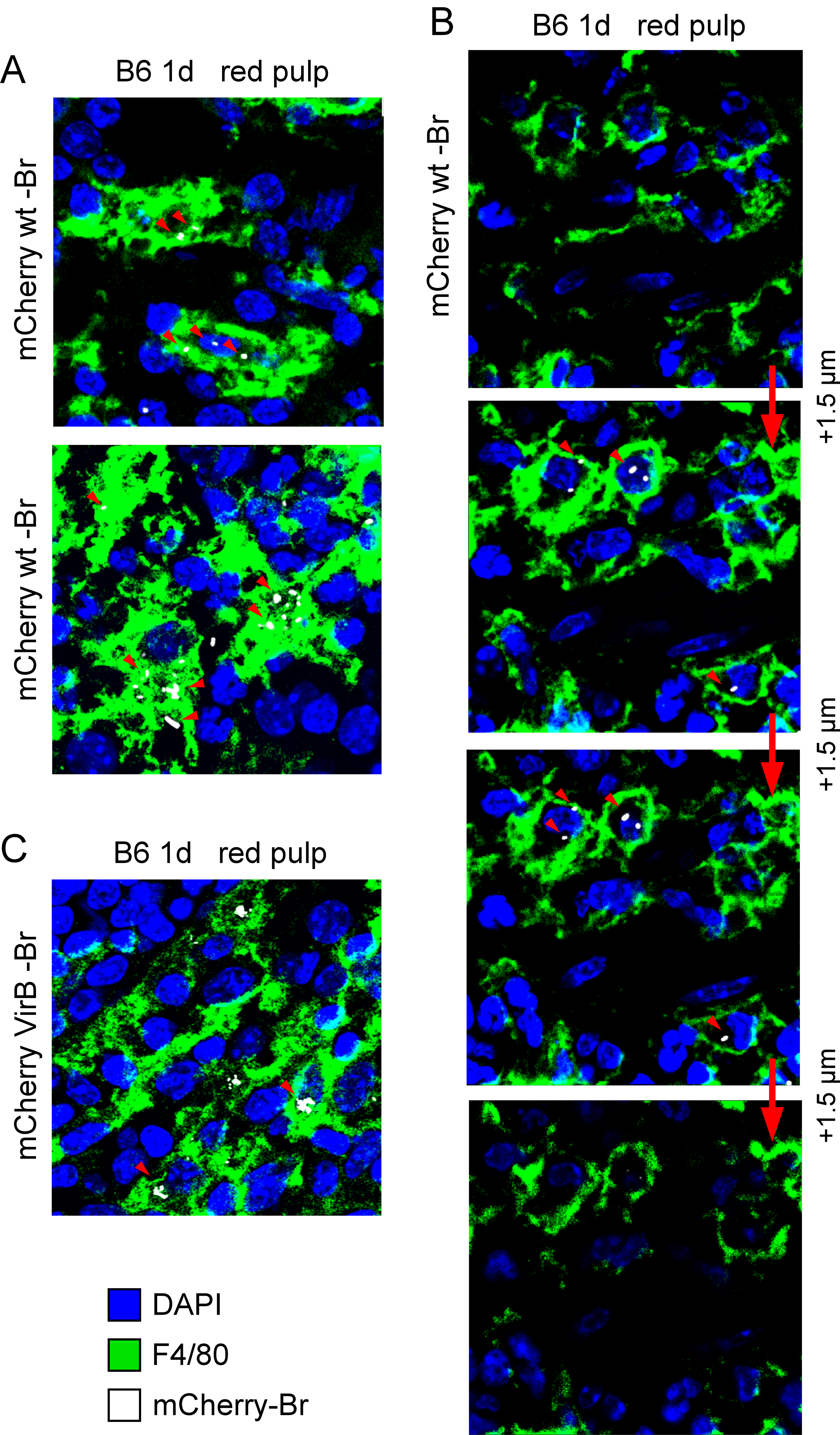

Supplement: Figure S5 — In vivo intracellular localization of Brucella determined by confocal microscopy. Wild-type C57BL/6 mice were injected i.p. with PBS or 108 CFU of mCherry-Br. Mice were sacrificed at 1 day p.i. and the spleens were collected and examined by confocal microscopy. A, Representative confocal images of spleen section infected with mCherry-Br and labeled with antibodies against F4/80 (green) antigen. B, Deconvolution process of a spleen section infected with mCherry-Br and labeled with antibodies against F4/80 (green) antigen. C, Representative confocal images of spleen section infected with mCherry-expressing ΔvirB B. melitensis mutant and labeled with antibodies against F4/80 (green) antigen. m, micrometer. (TIF) [file ppat.1002575.s005.tif]

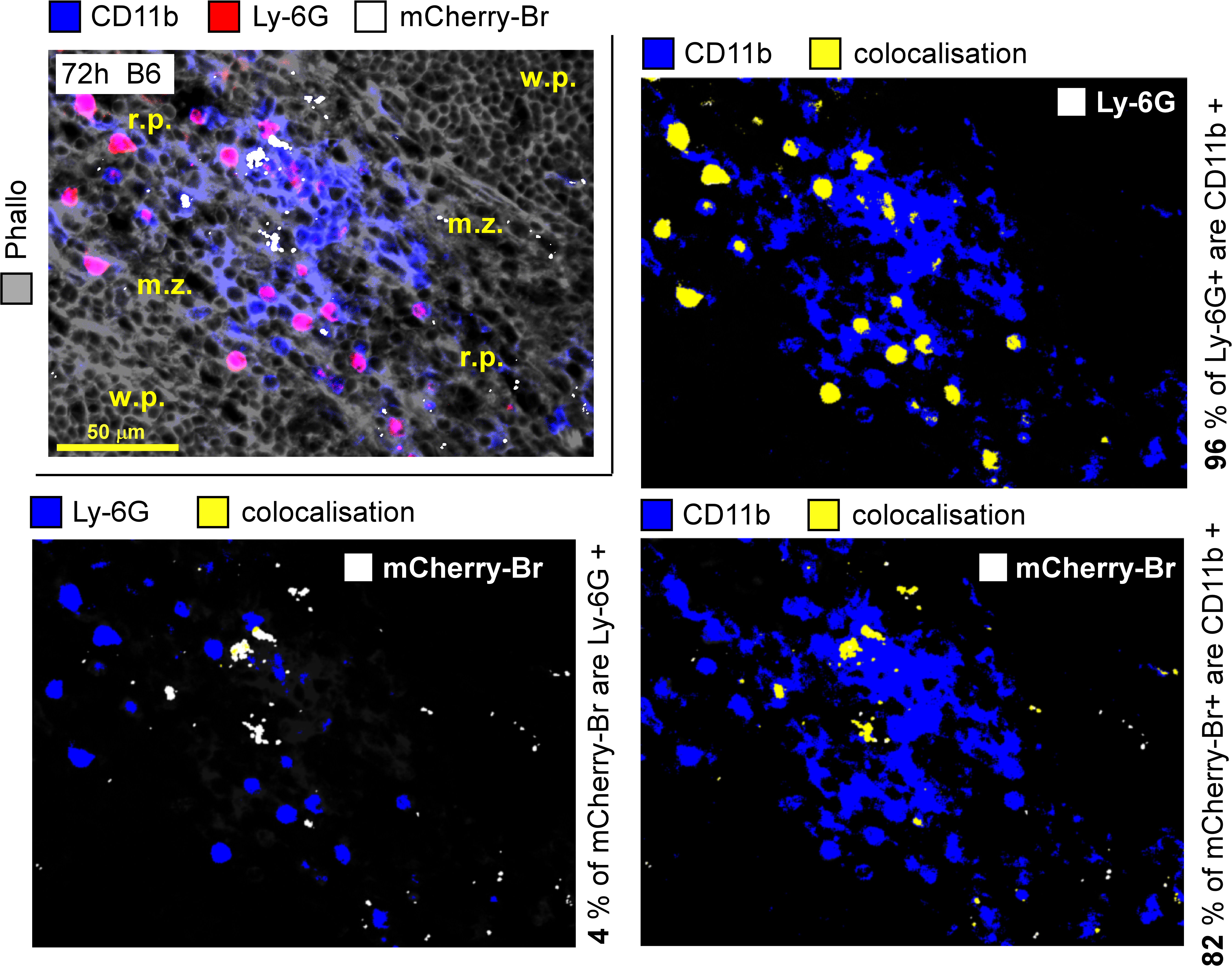

Supplement: Figure S6 — B. melitensis infects LY-6G−/CD11b+ during the course of infection. Colocalization assay of CD11b, Ly-6G expressing cells and mCherry-Br, 72 h after infection in wild-type C57BL/6 mice. Mice were injected i.p. with PBS or 108 CFU of mCherry-Br. Numbers indicate the percentage of colocalizing cells in the juxtaposing left panel. Panels are color-coded with the text for the antigen or mCherry-Br examined. Scale bar = 50 µm, as indicated. Data are representative of at least 3 independent experiments. r.p.: red pulp; w.p.: white pulp; m.z.: marginal zone. (TIF) [file ppat.1002575.s006.tif]

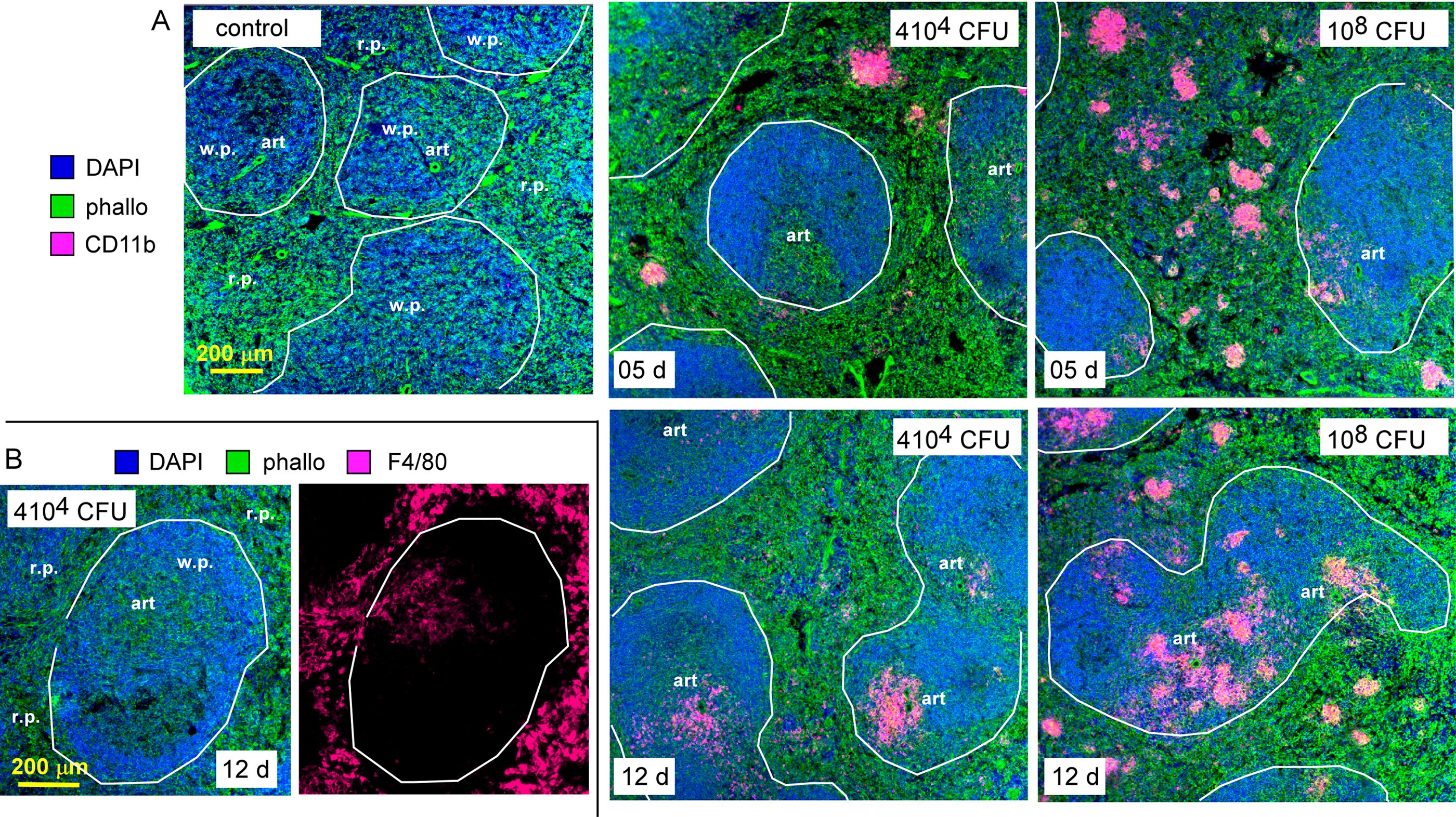

Supplement: Figure S7 — Granuloma formation in the spleen of mice infected by low and high doses of B. melitensis . Wild-type C57BL/6 mice (5 per groups) were inoculated i.p. with PBS, 4×10^4^ or 108 CFU of mCherry-Br, as indicated. At selected time, mice were sacrificed, spleens were collected and examined by immunohistofluorescence. Panels are color-coded with the text for the antigen examined (A), CD11b and (B) F4/80. Scale bar = 200 µm, as indicated. r.p.: red pulp; w.p.: white pulp; art.: central artery. Data are representative of 2 independent experiments. (TIF) [file ppat.1002575.s007.tif]

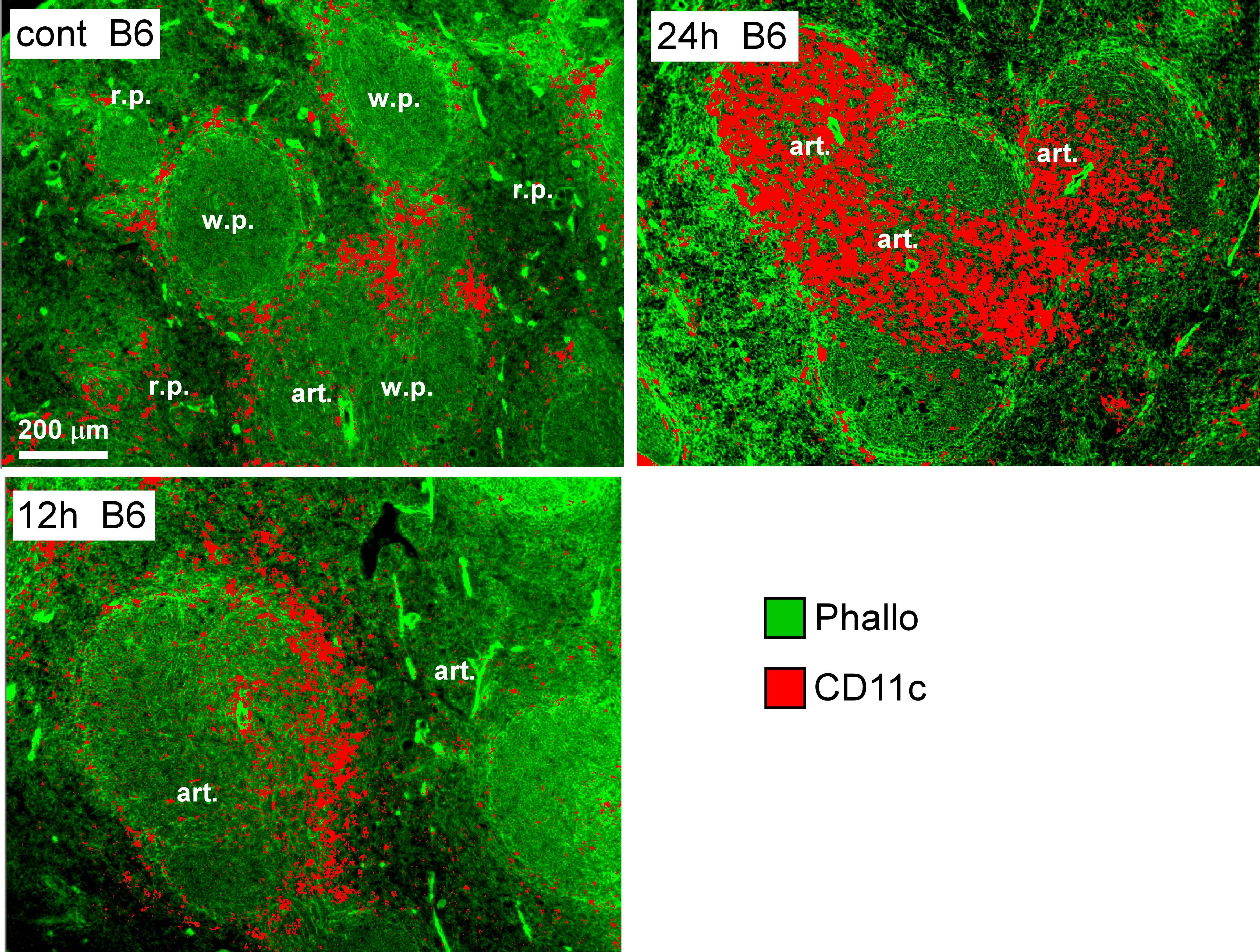

Supplement: Figure S8 — B. melitensis infection induces the DC migration in the spleen during the course of infection. Positioning of DCs in the spleen of mice before and 12 or 24 h after mCherry-Br inoculation. Wild-type C57BL/6 mice were injected i.p. with PBS or 108 CFU of mCherry-Br. Mice were sacrificed at selected times and spleens were collected and examined by immunohistofluorescence. Panels are color-coded with the text for the antigen examined. Scale bar = 200 µm, as indicated. r.p.: red pulp; w.p.: white pulp; art.: central artery. Data are representative of at least 3 independent experiments. (TIF) [file ppat.1002575.s008.tif]

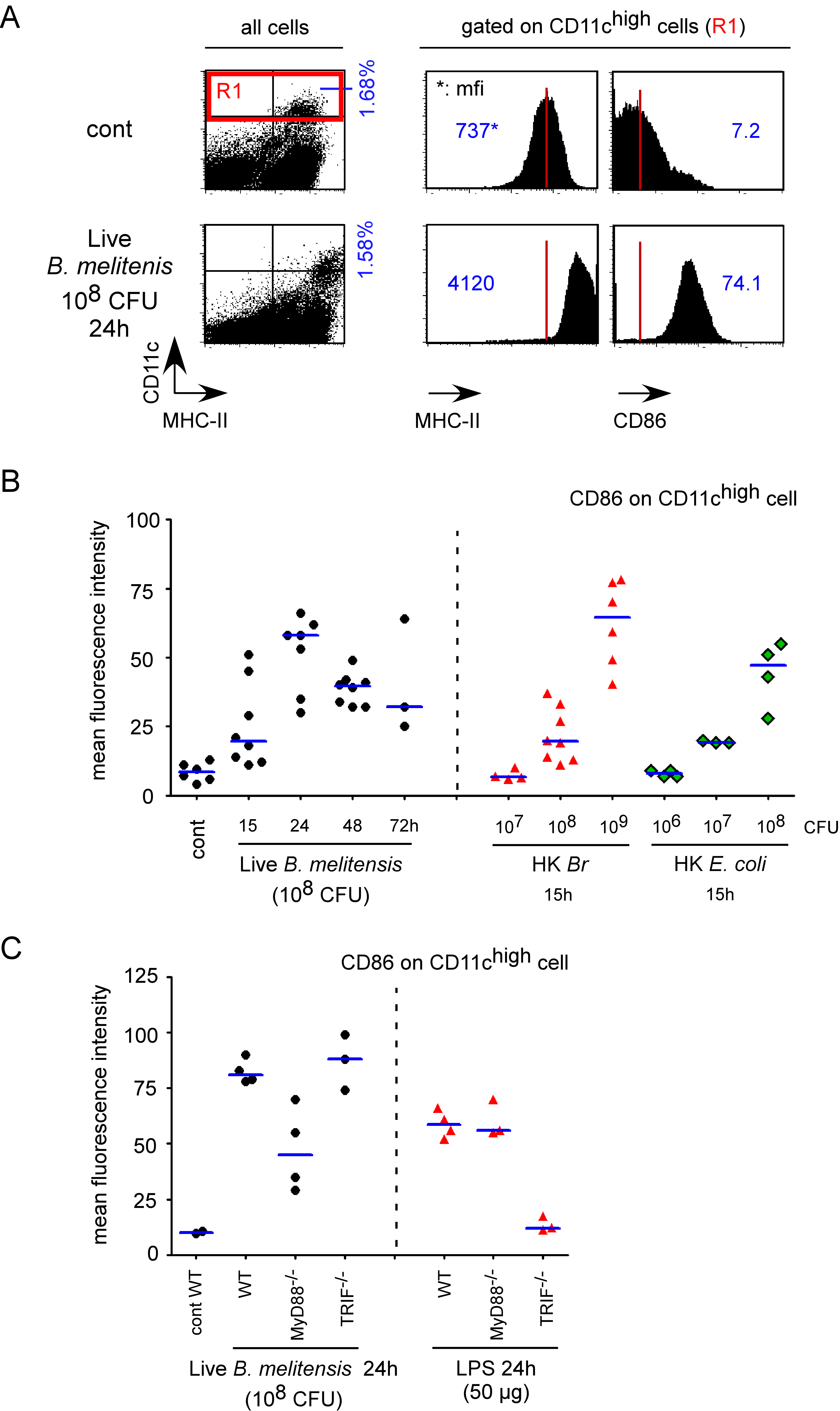

Supplement: Figure S9 — B. melitensis infection induces the DC maturation. Wild type, MyD88−/− and TRIF−/− C57BL/6 mice were injected i.p. with, 108 CFU of live B. melitenis and various doses of heat killed (HK) B. melitensis and Escherichia coli O18:K1. Mice were sacrificed at selected times and spleens were collected and analyzed by flow cytometry. Cells were gated according to size and scatter to exclude dead cells and debris from analysis. A, Spleen cells from individual mice were first analyzed for Forward Size Scatter (FSC) and CD11c expression. CD11chi cells in each group were then analyzed for MHC-II and CD86 expression. Number indicates the percentage of positive cells per 106 spleen cells acquired for the specified marker. B, C, Comparative analysis of CD86 level on CD11chi cells. The data are the median. Data are representative of at least 3 independent experiments. (TIF) [file ppat.1002575.s009.tif]

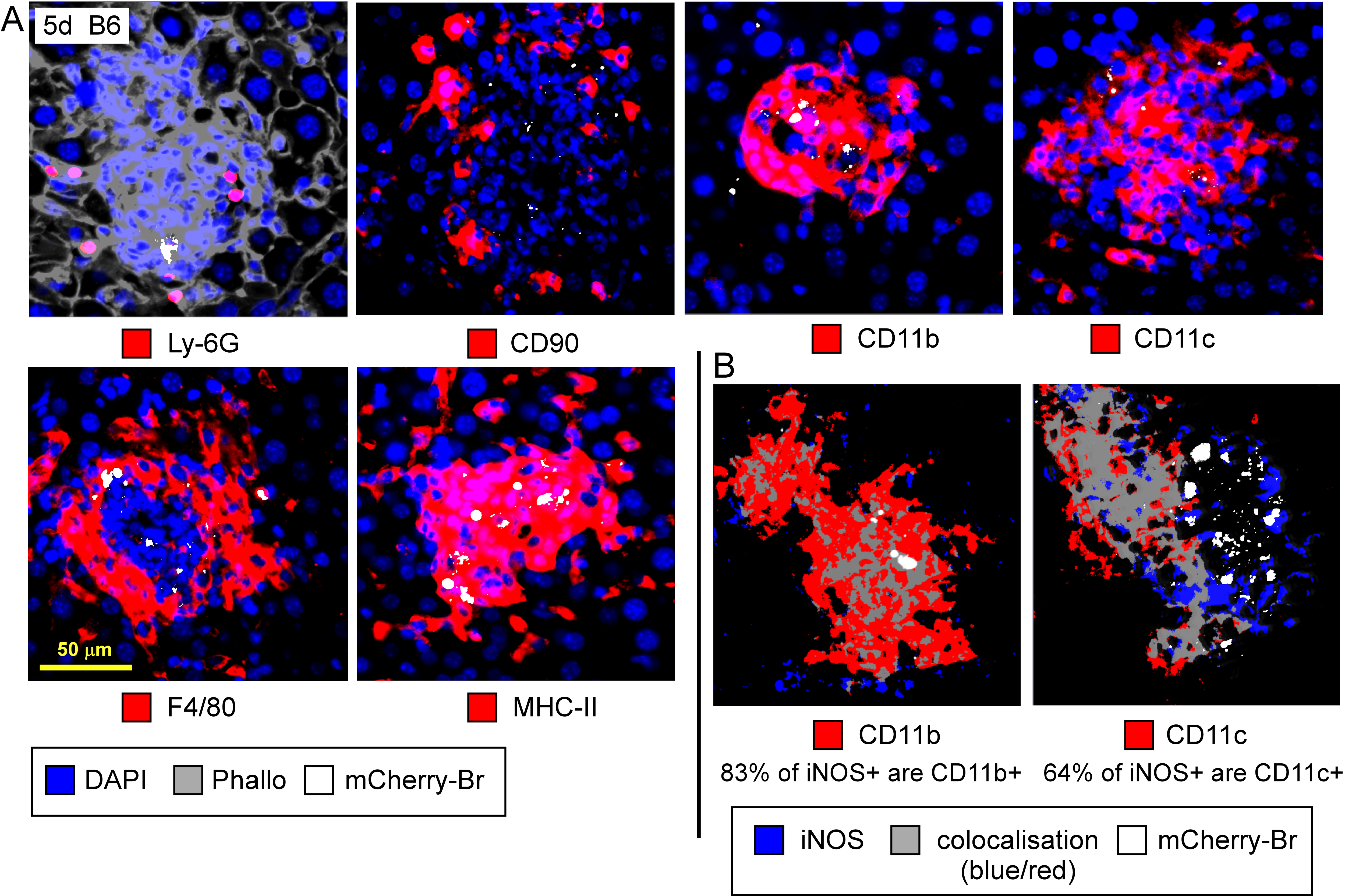

Supplement: Figure S10 — Phenotypical description of Brucella infected granuloma in the liver. Wild-type C57BL/6 mice were injected i.p. with PBS or 108 CFU of mCherry-Br. Mice were sacrificed at 5 days p.i. and livers were collected and examined by immunohistofluorescence. A, Immunofluorescence analysis of Ly-6G, CD90, CD11b, CD11c, F4/80, and MHC-II expressing cells and mCherry-Br. B, Percentage of iNOS+ cells that colocalizes with CD11b-, and CD11c-expressing cells. Images represent a single granuloma. Numbers in Figure B indicate the percentage of colocalizing cells in the upper panel. Panels are color-coded with the text for the antigen or mCherry-Br examined as well as the colocalization. Scale bar = 50 µm, as indicated. Data are representative of at least 3 independent experiments. (TIF) [file ppat.1002575.s010.tif]

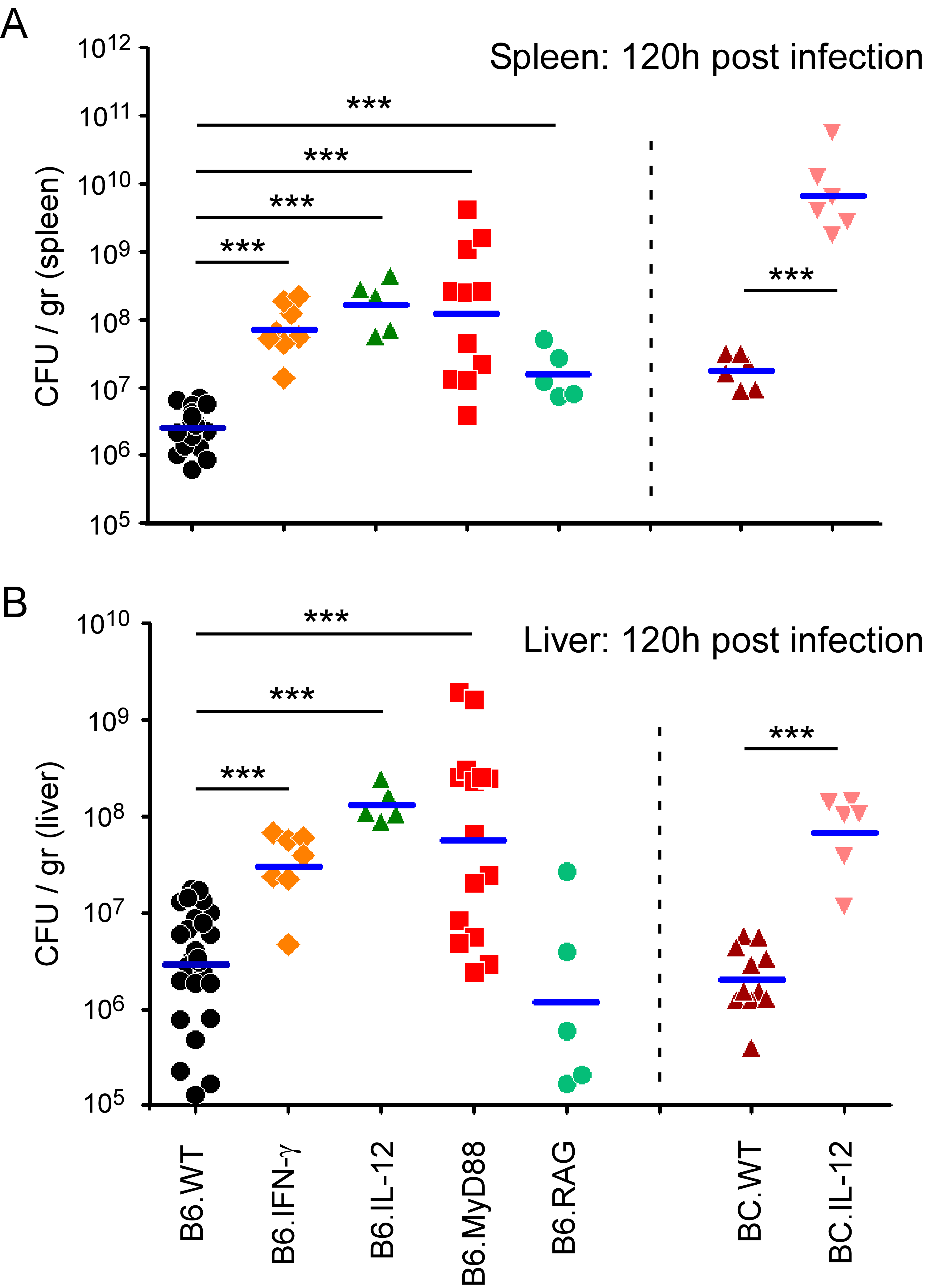

Supplement: Figure S11 — Analysis of the bacterial load of the spleen and liver in wild type and genetically deficient mice. Wild-type, IFN-γ−/−, IL-12p35−/−, MyD88−/− and RAG−/− C57BL/6 mice and wild-type and IL-12p40−/− BALB/c mice (3–6 per group) were inoculated i.p. with 108 CFU. 120 h p.i., CFU per g of spleen (A) and liver (B) were calculated. Data are pooled from 3 independent experiments displaying similar CFU mean for wild type mice. (TIF) [file ppat.1002575.s011.tif]

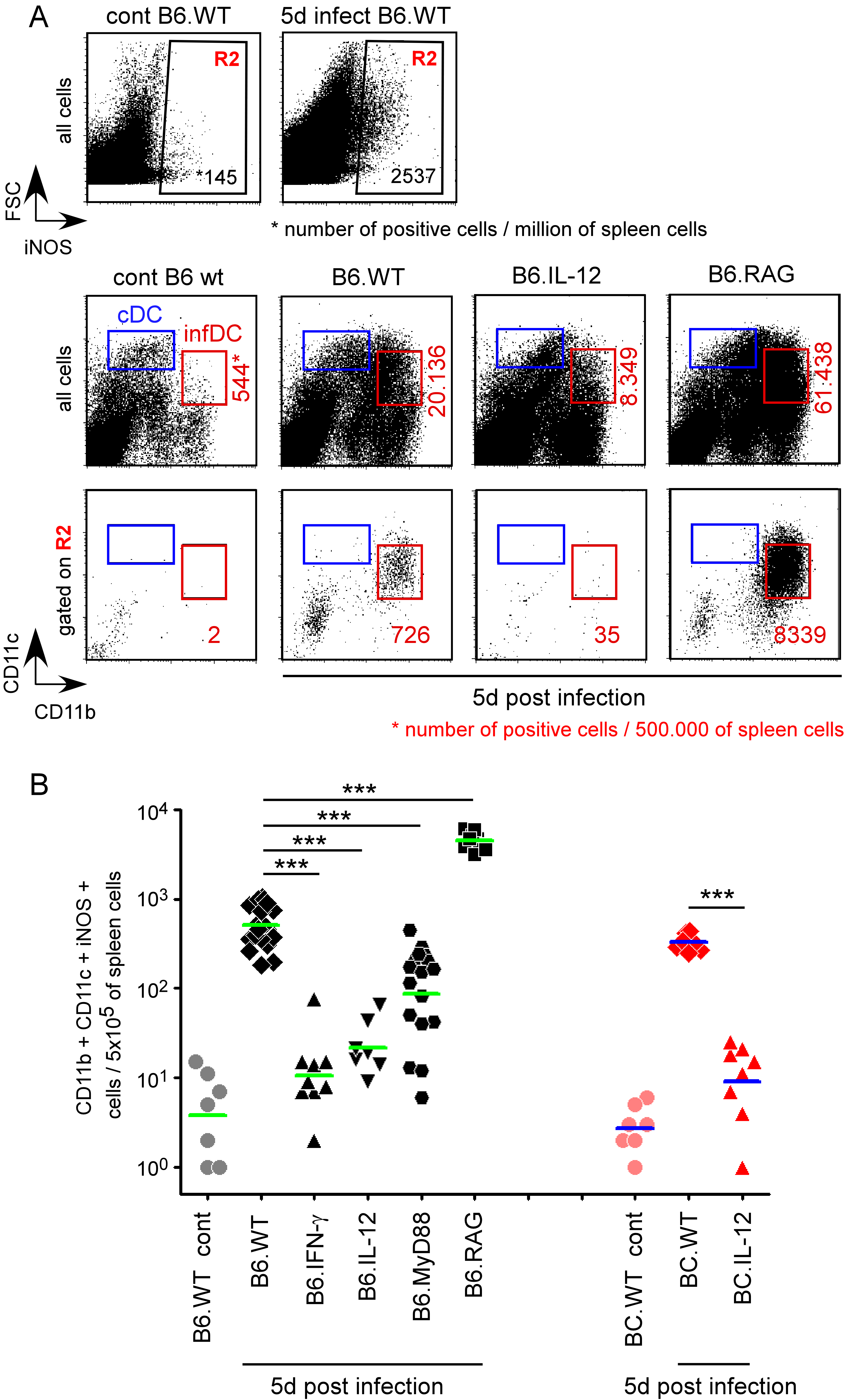

Supplement: Figure S12 — Flow cytometry analysis of iNOS-producing cells following B. melitensis infection. Wild-type, IFN-γ−/−, IL-12p35−/−, MyD88−/− and RAG−/− C57BL/6 mice and wild-type and IL-12p40−/− BALB/c mice (3–6 per group) were inoculated i.p. with 108 CFU of B. melitensis. Mice were sacrificed 5 days post-infection and spleen were collected and analyzed by flow cytometry. Cells were gated according to size and scatter to exclude dead cells and debris from analysis. A, Total pooled spleen cells were first analyzed for Forward Size Scatter (FSC) and iNOS expression. iNOS+ cells in each group were then analyzed for CD11b and CD11c expression. Number indicates the percentage of cells in the selected quadrant. B, Number of CD11b+ CD11c+ iNOS+ cells per 5×10^5^ spleen cells acquired. Each data represents the value obtained from an individual spleen and the data are pooled from 4 independent experiments. (TIF) [file ppat.1002575.s012.tif]

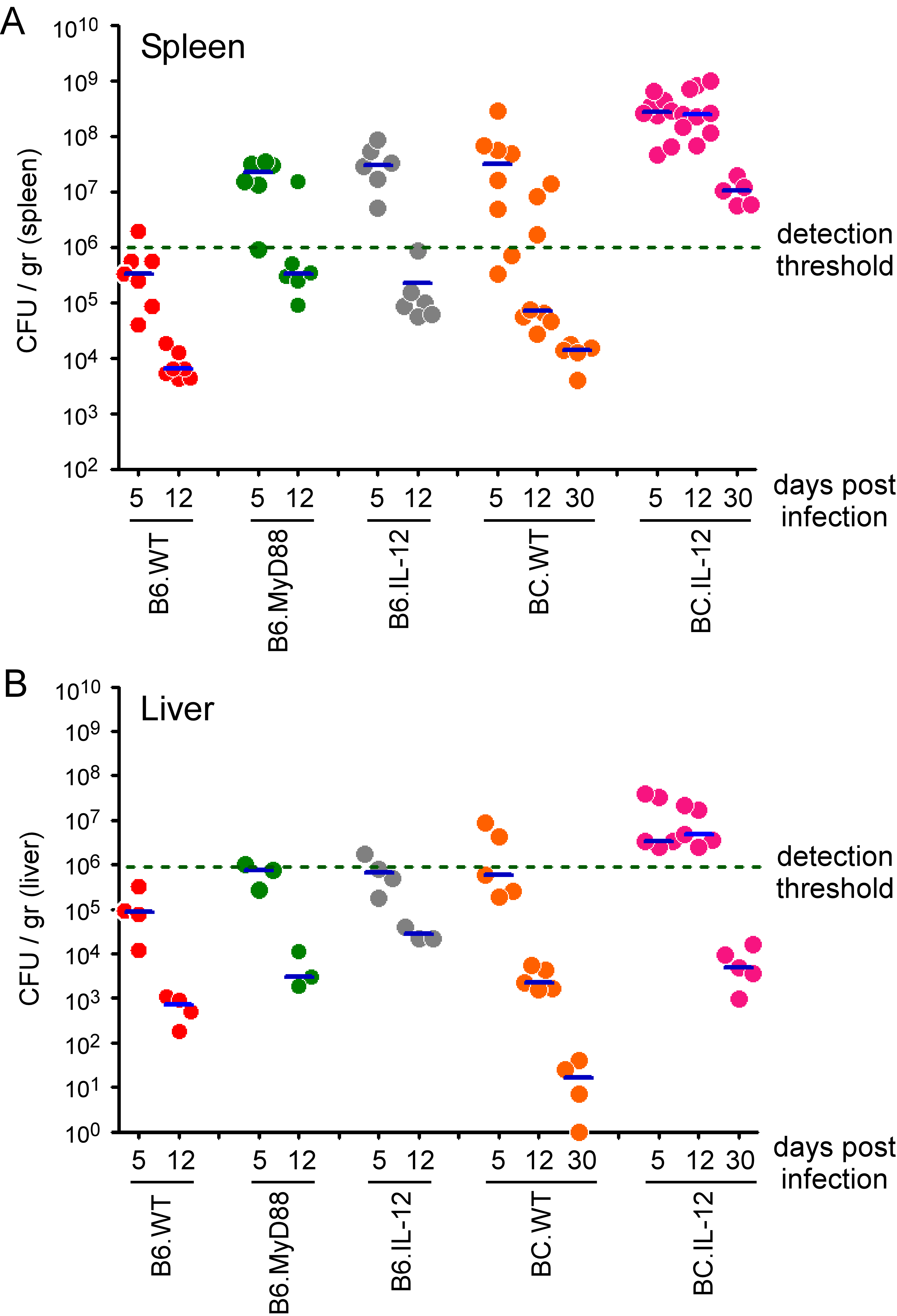

Supplement: Figure S13 — Kinetic analysis of the bacterial load in the spleen and liver in wild type and genetically deficient mice. Wild-type, MyD88−/− and IL-12p35−/− C57BL/6 mice and wild-type and IL-12p40−/− BALB/c mice (4–8 per group) were inoculated i.p. with 106 CFU. At 5, 12 and 30 days p.i., CFU per g of spleen (A) and liver (B) were calculated. Data are representative from 2 independent experiments. (TIF) [file ppat.1002575.s013.tif]

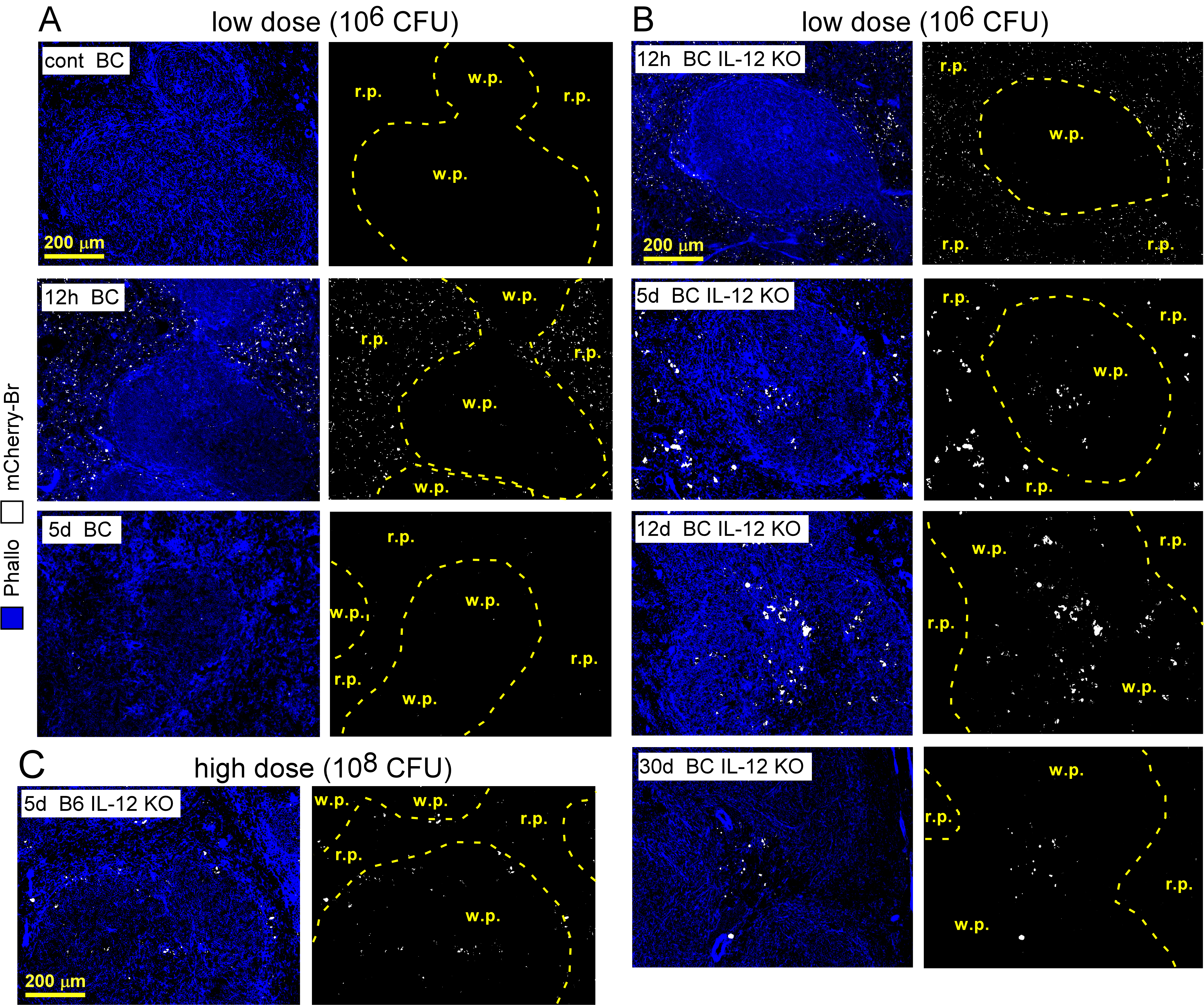

Supplement: Figure S14 — Localization of B. melitensis during the course of infection in wild type and Il-12-deficient BALB/c mice. Wild-type and Il-12p40−/− BALB/c and Il-12p35−/− C57BL/6 mice were injected i.p. with PBS, 106 CFU or 108 CFU of mCherry-Br, as indicated. Mice were sacrificed at 12 h, 5 days, 12 days, and 30 days p.i., as indicated. Spleens were collected and examined by immunohistofluorescence. Positioning of mCherry-Br in the spleen of Wild-type (A), Il-12p40−/− BALB/c (B) and Il-12p35−/− C57BL/6 mice (C). Panels are color-coded with the text for the antigen or mCherry-Br examined. Scale bar = 200, as indicated. r.p.: red pulp; w.p.: white pulp. Data are representative of at least 3 independent experiments. (TIF) [file ppat.1002575.s014.tif]
